# The Impact of Surface Chemistry and Synthesis Conditions on the Adsorption of Antibiotics onto MXene Membranes

**DOI:** 10.3390/molecules29010148

**Published:** 2023-12-26

**Authors:** Moyosore A. Afolabi, Dequan Xiao, Yongsheng Chen

**Affiliations:** 1 School of Civil and Environmental Engineering, Georgia Institute of Technology, Atlanta, GA 30332, USA; mafolabi3@gatech.edu; 2 Center for Integrative Materials Discovery, Department of Chemistry and Chemical & Biomedical Engineering, University of New Haven, West Haven, CT 06516, USA; dxiao@newhaven.edu

**Keywords:** MXene membrane, antibiotics, adsorption, water purification, density functional theory, 2D nanomaterials

## Abstract

MXene, a two-dimensional (2D) nanomaterial with diverse applications, has gained significant attention due to its 2D lamellar structure, abundance of surface groups, and conductivity. Despite various established synthesis methods since its discovery in 2011, MXenes produced through different approaches exhibit variations in structural and physicochemical characteristics, impacting their suitability for environmental application. This study delves into the effect of synthesis conditions on MXene properties and its adsorption capabilities for four commonly prescribed antibiotics. We utilized material characterization techniques to differentiate MXenes synthesized using three prevalent etchants: hydrofluoric acid (HF), mixed acids (HCl/HF), and fluoride salts (LiF/HCl). Our investigation of adsorption performance included isotherm and kinetic analysis, complemented by density functional theory calculations. The results of this research pinpointed LiF/HCl as an efficient etchant, yielding MXene with favorable morphology and surface chemistry. Electrostatic interactions and hydrogen bonding between MXene surface terminations and ionizable moieties of the antibiotic molecules emerge as pivotal factors in adsorption. Specifically, a higher presence of oxygen terminations increases the binding affinities. These findings provide valuable guidance for etchant selection in environmental applications and underscore the potential to tailor MXenes through synthesis conditions to design membranes capable of selectively removing antibiotics and other targeted substances.

## 1. Introduction 

In the last decade, there has been a growing exploration of MXene, a new, complex two-dimensional (2D) nanomaterial, driven by its unique properties. Due to its high aspect ratio, conductivity, and hydrophilicity, MXenes have been employed in a number of applications across a wide spectrum of research areas including energy storage, sensors, conductive coatings, and separation processes [1,2,3,4,5,6,7,8]. Similar to its predecessor graphene oxide (GO), MXene can be modified and assembled to make adsorbents, composite films, and membrane filters with relevance in environmental applications such as contaminant removal [6,9,10,11], water purification [12,13], and ion separation [7,8]. With its transition metal backbone and abundance of hydrophilic surface terminations, MXene bears chemical stability and functionality that positions it as a formidable competitor to GO in various environmental contexts. 

MXenes constitute a broad class of two-dimensional transition metal carbides and/or nitrides generated by selectively removing A-group elements from precursor MAX phases. Specifically, titanium carbide (Ti_3_C_2_T_x_) MXene has gained significant attention following its discovery in 2011 in which Ti_3_C_2_T_x_ was successfully derived by the selective etching of aluminum (an A-group element) from Ti_3_AlC_2_ powders, a MAX precursor, using hydrofluoric acid (HF) [14]. The resulting multilayer etching product can be delaminated to yield single- and few-layer nanosheets decorated with surface groups such as oxygen, hydroxyl, and fluorine (indicated by T_x_ in Ti_3_C_2_T_x_). Initial experimental procedures recommended the use of 50 wt. % HF etchants to remove aluminum layers [6,14]. However, further systematic refinements in synthesis showed that lower HF concentrations were equally effective in breaking the relatively weaker metallic bonds between aluminum and the Ti_3_C_2_ layers [15]. Moreover, excessive concentrations of HF were found to lead to MXenes with degraded morphology, an abundance of pinholes, and oxidation [16,17]. In situ HF etchant solutions created with fluoride salt/acid combinations such as lithium fluoride (LiF) with sulfuric (H_2_SO_4_) and hydrochloric acids (HCl) have emerged as established methods for successful MXene production [16,18,19]. Furthermore, alternative synthesis protocols have been developed to produce MXene materials, allowing for the desired control of surface terminations and morphology, catering to specific applications [20,21,22]. 

Given the critical role that the choice of etchant plays in shaping the resulting morphology and surface terminations of MXene materials, several studies have undertaken comprehensive investigations of the synthesis process, employing both theoretical and experimental approaches [22,23,24,25]. The intricate interplay between MAX precursors, etchant selection, acidic concentration, and precise etching duration can result in variations in surface chemistry [24,26]. This variability becomes particularly significant in applications where the surface chemistry profoundly influences the interlayer structure and provides essential sites for further modifications. Such considerations hold particular relevance for MXene films and filters, where the surface terminations hold equal importance for adsorption and the retention of contaminants susceptible to ion exchange, charge–charge interactions, and van der Waals forces [6,11,27]. In membrane filtration, regardless of the fabrication structure (free-standing films or supported films), the adsorptive interactions between the MXene surface terminations and target contaminants such as dyes and pharmaceuticals, in addition to molecular sieving, are attributed to the overall removal mechanism. Indeed, the unique performance of MXene membranes is largely attributed to their surface functionality, to the extent that the adsorption of antibiotics of similar size and class can be differentiated based on electrostatic interactions [11]. Since the complexity of MXene surface terminations is predominantly dictated by the synthesis formulation, it is of importance to explore the effects of straightforward synthesis conditions on the resulting properties of MXene materials and the film adsorption capability. This exploration is crucial for understanding the subsequent implications on the synthesis–property–performance relationships of MXene in various environmental applications. 

In this study, we systematically evaluated MXene materials synthesized via three common prevalent methods—HF etchant, HCl/HF etchant, LiF/HCl etchant—for their applicability in environmental contexts. Our investigation began by examining the morphological and elemental distinctions among the resulting MXene variants through comprehensive material characterization. Subsequently, we employed these MXenes to fabricate active layer films, where their potential adsorption and separation were rigorously assessed using a model system involving the adsorption of four commonly encountered antibiotics. Our analysis encompassed adsorption isotherm and kinetic studies, complemented by density functional theory (DFT) calculations. These efforts have been undertaken to unveil the intricate relationship between surface terminations derived from the synthesis process and their consequential impact on adsorption performance. 

## 2. Results and Discussion 

### 2.1. Ti_3_C_2_-MXene Characterization

**Figure 1:**
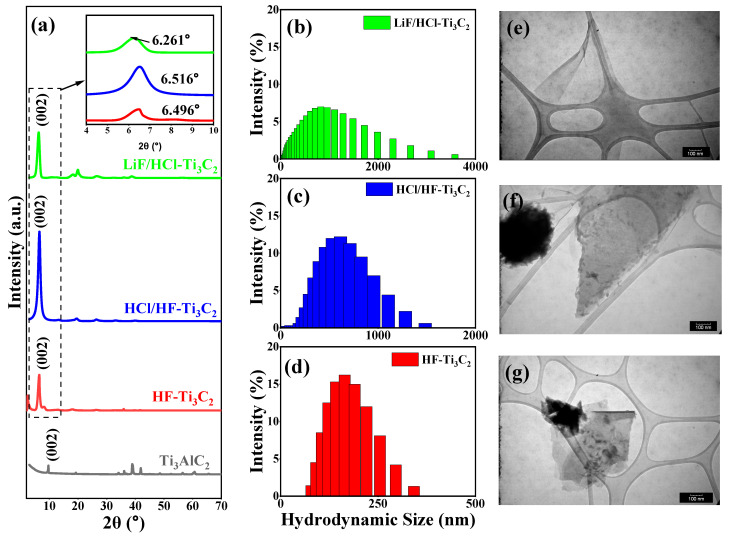
(**a**) XRD spectra of LiF/HCl-Ti_3_C_2_, HCl/HF-Ti_3_C_2_, and HF-Ti_3_C_2_ films with (002) peaks for each MXene displayed in the inset plot, (**b**–**d**) hydrodynamic size measurements of MXene nanosheets taken via dynamic light scattering, (**e**–**g**) TEM images of MXene nanosheets, captured on a 300-carbon mesh grid, with a scale bar = 100 nm.

The three methods of chemical etching applied to Ti_3_AlC_2_ were successful in producing the MXenes, HF-Ti_3_C_2_, HCl/HF-Ti_3_C_2_, LiF/HCl-Ti_3_C_2_, all with varying characteristics. Evidence confirming the effective removal of aluminum layers through both in situ and explicit hydrofluoric acid etching could be gleaned from the XRD measurements (Figure 1). Specifically, the characteristic peak (002) shifted from 9.669° (with a d-spacing = 9.140 Å) in the precursor Ti_3_AlC_2_ to lower Bragg angles of 6.496°, 6.516°, and 6.261° for HF-Ti_3_C_2_, HCl/HF-Ti_3_C, and LiF/HCl-Ti_3_C, respectively. These expansions in the d-spacing resulted from the loss of aluminum and the subsequent delamination facilitated by sonication and Li^+^ intercalation, particularly notable in the cases of the HCl/HF and LiF/HCl etchants [28]. Among these, the largest d-spacing observed was 14.105 Å for LiF/HCl-Ti_3_C_2_, while HF-Ti_3_C_2_ and HCl/HF-Ti_3_C_2_ exhibited slightly smaller d-spacings of 13.596 Å and 13.554 Å, respectively. However, it is worth noting that HF-Ti_3_C_2_, while effective in etching, led to the production of smaller MXene flakes, as evident from both the TEM imaging (Figure 1d) and dynamic light scattering measurements (Figure 1g). This finding has been previously attributed to the high hydrofluoric acid content and prolonged sonication times required during HF-Ti_3_C_2_ etching [17,29,30]. As a result, mixed acid etching and fluoride salt etching such as HF/HCl and LiF/HCl employed in this work have become the preferred method out of the synthesis methods [15,18,24]. Interestingly, these methods yielded larger MXene sheets (Figure 1) with average lateral dimensions of 743.85 nm for HCl/HF-Ti_3_C_2_ and 902.07 nm for LiF/HCl-Ti_3_C_2_, respectively.

X-ray photoelectron spectroscopy (XPS) survey scans and narrow region scans encompassing the C 1s, Ti 2p, O 1s, and F 1s regions provided insights into the composition and chemical states of the synthesized MXene. Notably, aluminum was absent in all survey spectra, further indicating a successful etching process. While the chemical composition of the MXenes appeared similar on the surface, a more detailed analysis involving depth profiling over a 400 µm spot size with Ar^+^ sputtering revealed differences in atomic compositions in the near-surface interior (refer to Figure S1). The respective elemental compositions generally remained consistent across the various etched layers (50, 100, and 150 s), and the average of the spectra obtained from 0 to 150 s are summarized in Table 1. Of note, LiF/HCl-Ti_3_C_2_ exhibited the highest oxygen composition at 20.99%, whereas HCl/HF-Ti_3_C_2_ had the highest O/F ratio, reaching 2.68. HF-Ti_3_C_2_ contained the highest fluorine content, attributed to its formulation with a 30 wt.% HF solution, while the other two MXenes were formulated with an effective concentration of 5 wt.% HF solution, resulting in a significantly lower fluorine content. These variations in composition arising from different synthesis conditions align with findings from previous studies [17,18]. Noticeably, HF-Ti_3_C_2_ displayed a higher carbon composition across both the surface and interior. This could be attributed to excess etching, a higher presence of potential contamination, and the presence of adventitious carbon [31].

**Figure 2:**
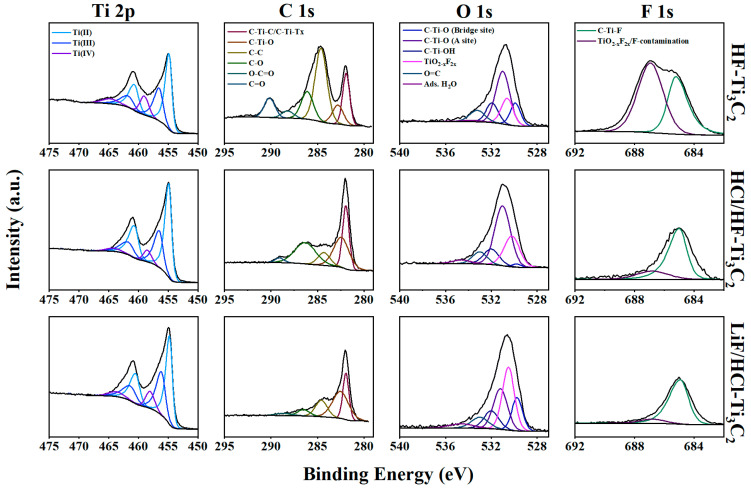
XPS spectra of HF-Ti_3_C_2_, HCl/HF-Ti_3_C_2_, and LiF/HCl-Ti_3_C_2_ (etch time = 50 s).

Peak fitting of narrow region scans offers a more detailed understanding of the speciation of relevant MXene structure moieties. In our discussion, we considered the spectra produced from a 50-s etch-time to represent the near-surface interior (Figure 2). While various methods have been proposed for fitting the Ti 2p region [17,32,33], our approach in this study was based on the oxidation state and chemical environment, following the recommendation by Varun et al. [32]. The Ti 2p region had two main peaks due to spin-orbit splitting: 2p_3/2_ and 2p_1/2_ with energy levels around 455 eV (ca.) and 460.5 eV (ca.), respectively. Within these peaks, we identified two relevant MXene component doublet peaks based on their 2p_3/2_ peak: Ti^2+^ at ca. 455 eV and Ti^3+^ at ca. 456 eV. An additional doublet peak was attributed to Ti^4+^, which represented a partially oxidized degradation product of TiO_2-x_F_x_, appearing at ca. 459 eV. Specific binding energy, full width at half maximum values, and atomic percentages for each MXene type can be found in Tables S2–S7, covering both Ar^+^ etched spectra and non-etched spectra.

For all MXenes, most of the titanium could be attributed to Ti^2+^, with average oxidation states of MXene-Ti being 2.38, 2.36, and 2.39 for HF-Ti_3_C_2_, HCl/HF-Ti_3_C_2,_ and LiF/HCl-Ti_3_C_2_, respectively. These findings aligned with previous research [34]. Additionally, we applied another method of peak fitting to estimate the surface terminations by fitting different chemical octahedra that could potentially surround titanium including C-Ti-O/O/O, C-Ti-O/O/F, C-Ti-O/F/F, and C-Ti-F/F/F. These peaks were fitted in increasing binding energy order based on the average electronegativity of the surface terminations. As such, C-Ti-O/O/O was fitted at ca. 455 eV, while C-Ti-F/F/F was fitted at ca. 458 eV (refer to Table S8 and Figure S3). This peak fitting revealed that most chemical environments could be attributed to O/O/O termination for all MXenes. However, HF-Ti_3_C_2_ exhibited a relatively larger presence of C-Ti-F/F/F and co-adsorbed terminations (C-Ti/O/O/F and C-Ti-O/F/F) exclusively on its surface (at 0 s etch-time) in comparison to the other MXenes (Figure S3). This suggests that oxygen may be the favored termination for satisfying surface energy following the removal of the aluminum atoms during etching. This observation is in line with the theoretical calculations [25], although previous experimental work has also shown that a higher HF wt.% in the etching process results in a lower O/F ratio and increased -F terminations [35]. While HF-Ti_3_C_2_ may have high fluorine, peak fitting supported that most of the fluorine terminations may be co-adsorbed with oxygen, rather than full fluorine termination (C-Ti-F/F/F) in the chemical octahedra. Additionally, the peak fitting analysis suggests that a higher HF concentration also led to a higher fraction of TiO_2-x_F_2x,_ which is a product of oxidation and degradation due to the more aggressive etching condition. 

In the C 1s region spectra, we performed fitting with two main MXene components representing carbon within a titanium carbide skeleton at ca. 282.0 eV and titanium carbide bonded to oxygen termination at ca. 282.5 eV. Additionally, we accounted for other components related to adventitious carbon and contaminations within the spectra including C–C at ca. 284.8 eV, C–O at ca. 286.2 eV, O–C=O at ca. 288.8 eV, and C=O at ca. 290 eV. Notably, LiF/HCl-Ti_3_C_2_ exhibited the most prominent MXene peaks in its C 1s region, with a high fraction of 0.69. HCl/HF-Ti_3_C_2_ had a slightly lower fraction at 0.57, while HF-Ti_3_C_2_ displayed a distinguishable, but lower fraction of titanium carbide of 0.27. This difference could be the result of the high presence of captured carbon adsorbates and the formation of graphitic and amorphous carbon, which can occur with high oxidation due to the harsh etching conditions [36]. 

In the O 1s narrow scan region, a broad peak was observed, comprising several MXene and non-MXene components that could be fitted. Based on previously reported studies, MXene O 1s peaks including C-Ti-O (bridge site), C-Ti-O (A site), and C-Ti-OH, were centered at ca. 529.8 eV, 531.0 eV, and 532 eV, respectively [17,32]. The oxygen at the bridge site has been theorized to be located above and between the nearest titanium and carbide atoms, while oxygen on the A site refers to oxygen located parallel to the interior titanium [37]. Additional peaks corresponding to the oxidation/degradation product TiO_2-x_F_2x_ were assigned to ca. 530.6 eV, along with peaks related to organic contamination and adsorbed water molecules. Across the three MXene types, the C-Ti-O component constituted the majority of the area under the broad spectra. Without Ar^+^ sputtering, the majority of oxygen in C-Ti-O was positioned at the bridge site. However, this majority shifted to the A site in the near-surface interior and bulk, as supported by a previous study by Persson et al., which found this specific coordination to be thermodynamically favorable [38]. Persson et al. also noted in their theoretical work that oxygen on the A site could also be influenced by co-adsorbed fluorine, shifting the A-site oxygen peak to a higher binding energy. This observation was confirmed in our analysis, where LiF/HCl-Ti_3_C_2_ exhibited a higher binding energy estimated for the A-site peak (531.2 eV) compared to HCl/HF-Ti_3_C_2_ and HF-Ti_3_C_2_ (both at 531.0 eV). The presence of co-adsorbed oxygen and fluorine termination was also supported by peak fitting in the Ti 2 p region, which assisted in visualizing the surface composition of the MXenes. Among the three MXenes, the fraction of C-Ti-OH in the O 1s spectra was estimated to be highest (0.22) for HF-Ti_3_C_2_ on the surface without sputtering. However, with sputtering, similar fraction amounts of C-Ti-OH were found throughout the bulk materials, ranging between 0.12 for LiF/HCl-Ti_3_C_2_ and 0.14 for HF-Ti_3_C_2_. It is worth noting that XPS was conducted under vacuum conditions, where hydroxyl surface terminations should be desorbed, implying that XPS quantification may not accurately estimate the presence of the hydroxyl terminations. Nevertheless, the presence of hydroxyl groups is intuitively expected to be higher at low pH when oxygen terminations are protonated and much lower at high pH when the oxygen group deprotonated, which becomes relevant in the adsorption experiments conducted in this work. Finally, the degradation by-product TiO_2-x_F_2x_ was observed in all MXenes, centered around 530.5–530.8 eV.

In the F 1s region, the primary MXene component observed was C-Ti-F, centered around 685 eV. This characteristic peak was present in all MXene samples. Additional fluorine peaks could be attributed to F co-adsorbed in TiO_2-x_F_2x_ and other fluorine contamination including organic and unidentified species. Notably, HF-Ti_3_C_2_ exhibited a higher content of F-contamination, which might manifest in the form of fluorinated polymeric carbon chains and other unknown fluorinated compounds resulting from the harsh etching process. 

In contrast to MXenes produced with lower HF content, HF-Ti_3_C_2_ exhibited structural deficiencies. While the XRD measurements of the dry MXene films exhibited a relatively smaller d-spacing for HF-Ti_3_C_2_, the measurements of wet films revealed a notably large interlayer spacing after immersion in water (Table S1). A previous study involving characterization and structural modeling demonstrated that MXenes with a significantly higher presence of one functional group over others such as a substantial excess of -F relative to –O or –OH as seen in in HF-Ti_3_C_2_ could easily disrupt interlayer stacking [35]. The disproportionate presence of one termination could reduce the likelihood of hydrogen bonding between –OH and –F or –O, while increasing the probability of repulsion between like terminations (F–F, O–O). This disorderly interlayer stacking can be disturbed when polar molecules such as water intercalate between the nanosheets. As both HF-Ti_3_C_2_ and HCl/HF-Ti_3_C_2_ had high concentrations of either fluorine or oxygen (O/F ratios of 0.54 and 2.68, respectively), a greater degree of swelling could occur, in comparison to the swelling of LiF/HCl-Ti_3_C_2_ (Table S1). Additionally, HF-Ti_3_C_2_ had a significantly higher presence of non-MXene related carbon, specifically C-C, as indicated by XPS characterization. This is consistent with previous studies that used high HF content [6,39]. Excess HF may not only target the relatively weaker metallic bonded aluminum layers in the MAX phase, but may also attack the titanium carbide bond, resulting in the formation of amorphous carbon and other carbonates (C=O, CF_2,_ etc.). Surface moieties had a clear impact on the resulting Mxene, as shown by the characterization in this work and previous studies [17,31,35]. In both 2D and 3D applications such as membrane filters and adsorbents, the stacking structure and available surface terminations could affect important removal mechanisms including adsorption. The effect of adsorption will be investigated in the following section.

### 2.2. Antibiotic Adsorption Isotherm Investigation 

Given that each MXene synthesized from different etching conditions exhibited varying chemical and morphological characteristics, these synthesis-derived features have the potential to define and affect the interactions between contaminant molecules and MXene film surfaces. To assess these distinctions, batch adsorption experiments were conducted using the as-synthesized MXenes, with four different antibiotics as model contaminants: levofloxacin (LEV), norfloxacin (NOR), trimethoprim (TMP), and sulfamethoxazole (SMX) (relevant characteristics are defined in Table 2). These antibiotics were chosen due to their different molecular weight, charge, and structures, which represent a range of potential pharmaceutical contaminants in these adsorption experiments. Each synthesized MXene film was immersed in antibiotic solutions with various initial concentrations for 24 h, after which the equilibrium concentrations were determined through liquid chromatography analysis, as described in analytical methodology section. The relationship between adsorbed mass (q_e_) and equilibrium concentration in the liquid phase (C_e_) was assessed by developing adsorption isotherms (Freundlich, Langmuir, and Dubinin–Radushkevich) through regression analysis based on the adsorption isotherm equations. The Freundlich isotherm model was determined to be a more suitable model to describe the adsorption process than the Langmuir model based on the results of both linear and nonlinear curve fitting (See Tables S10–S14). Table 3 summarizes the Freundlich parameters (K_F_ and *n*) and model statistical parameters (root mean square error, RMSE; additional fitting results are found in Tables S10–S14). While the Langmuir isotherm model describes monolayer adsorption, the Freundlich isotherm encompasses multilayer adsorption on a heterogenous adsorbent surface with an exponential distribution of active sites and adsorption energies [40]. This implies that all MXene-composite membranes have a variety of active sites with different binding affinities toward each of the antibiotic molecules. The adsorption capacity of each MXene could be roughly indicated by parameter K_F_. In comparison to other MXene composites, LiF/HCl-Ti_3_C_2_ generally exhibited the highest adsorption capacity. However, the adsorption performance of the MXenes varied across antibiotics. NOR on LiF-Ti_3_C_2_ displayed the highest K_F_ value (K_F_ = 0.498 (mg/g)/(mg/L)^n^), followed by NOR on HCl/HF-Ti_3_C_2_ (0.346 (mg/g)/(mg/L)^n^). LEV also showed high adsorption on LiF/HCl-Ti_3_C_2_ (0.228 (mg/g)/(mg/L)^n^). Both NOR and LEV belong to the fluoroquinolone antibiotic class but differ in molecular weight due to the additional aromatic ring and methyl group in LEV. At neutral pH, both NOR and LEV are zwitterionic, although NOR carries a slightly positive charge due to the protonated amine group, while LEV carries a slightly negative overall charge with the positive amine group hindered by a methyl group. This difference may contribute to the lower K_F_ values for LEV with the negatively charged LiF-Ti_3_C_2_ surface. NOR and LEV exhibited favorable adsorptions for all MXene surfaces, as indicated by values for *n* that were all above 1 (Table 3). 

TMP, a small positive antibiotic molecule, exhibited favorable adsorption with LiF/HCl-Ti_3_C_2_ (*n* = 1.486) and HCl/HF-Ti_3_C_2_ (*n* = 1.159) and K_F_ values of 0.197 (mg/g)/(mg/L)^n^, 0.161 (mg/g)/(mg/L)^n^, respectively. These values were somewhat comparable to those of LEV on MXenes, implying that the positive charge of TMP significantly contributes to its adsorption. Conversely, SMX, a smaller, strongly negatively charged antibiotic molecule, exhibited low adsorption capacity with all MXene adsorbents, and the adsorption was deemed unfavorable based on the K_F_ and *n* values. Adsorption isotherm fits for the antibiotics on PVDF alone showed that Freundlich was also a good fit to describe the adsorption process. However, the Freundlich constants suggested that there was minimal adsorption on PVDF, except for TMP (K_F_ = 0.103 (mg/g)/(mg/L)^n^), probably due to its strong positive charge and small size, which facilitated some degree of adsorption on the PVDF surface. The PVDF membrane also had significantly lower surface functionality (oxygen groups) compared to the MXene active layer based on the XPS analysis (see Table 1). The lack of surface groups could greatly reduce potential adsorption sites for the antibiotics. In previous investigative works, the low adsorption of SMX and other sulfonamide antibiotics on plastics and similar polymer membranes (polyamide and polyvinyl chloride) has been demonstrated [41,42]. These studies determined that the adsorption of SMX was also reasonably modeled by the Freundlich isotherm, and low uptake was attributed to the small number of hydrophilic sites on the polymer for the relatively hydrophilic SMX molecule to adsorb. In comparison, TMP is relatively more hydrophobic (as described by partition coefficients, Log D = 0.92 and 0.03 for TMP and SMX, respectively; see Table S9) and has previously been shown to have better adsorption to polyamide thin film active layers than SMX [42] (comparative Freundlich parameter values are compiled in Table S18). Furthermore, the concave curvature of the isotherm fit indicated that the adsorption process on PVDF was primarily driven by adsorbate–adsorbate interactions rather than adsorbent–adsorbate interactions in the case of antibiotic-PVDF adsorption [40]. 

**Table 2:** Summary of antibiotics and their respective characteristics including chemical structures visualized using VESTA [43]. Molecular weight, projected radii, pK_a_, and charge information were computed using ChemAxon’s Chemicalize [44].

Antibiotics	Chemical Structure	Molecular Weight (g/mol)	Projection Radius (Å) (Min/Max)	pK_a_	Charge pH 2.8	Charge pH 7	Charge pH 11.5
Sulfamethoxazole (SMX)	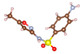	253.28	5.40/5.88	1.97, 5.86	+0.16	−0.93	−1.00
Trimethoprim (TMP)	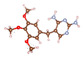	290.32	4.97/6.95	0.10, 7.16	+1.00	+0.93	+0.00
Norfloxacin (NOR)	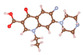	319.34	5.16/7.44	5.97, 8.69	+1.00	+0.14	−0.99
Levofloxacin (LEV)	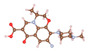	361.37	5.27/8.20	5.77, 8.31	+0.99	−0.06	−0.99

Additionally, the Dubinin–Radushkevich isotherm fitting was performed to estimate the sorption free energy (E, kJ/mol) for each adsorbate–adsorbent scenario (parameter values listed in Table 3). Although NOR exhibited the highest calculated sorption energies, all of the calculated values were below 8 kJ/mol, indicating that physisorption was the dominant adsorption mechanism [40]. This implies that the multilayer adsorption between the antibiotic molecules and MXene surfaces is facilitated by van der Waals forces, electrostatic interactions, and hydrogen bonding, rather than chemical bonding. Furthermore, LiF/HCl-Ti_3_C_2_ had comparatively higher sorption energies. The sorption energy was calculated to be 1.34 kJ/mol and 0.736 kJ/mol for NOR and LEV, respectively, on LiF/HCl-Ti_3_C_2_ in comparison to 0.357 kJ/mol and 0.532 kJ/mol for NOR and LEV, respectively, on HF-Ti_3_C_2_. The results of the adsorption isotherms point at the increased physisorption ability of the MXenes synthesized with lower HF content (HCl/HF-Ti_3_C_2_ and LiF/HCl-Ti_3_C_2_) in comparison to the MXene synthesized at higher HF content (HF-Ti_3_C_2_), in addition to highlighting the potential influence of the antibiotic structure and charge on the adsorption process.

Previous studies have also found the adsorption of organic contaminants on 2D nanomaterials to follow the Freundlich isotherm model. Jun et al. investigated the adsorption of methylene blue (MB, 319.85 g/mol), a positively charged dye molecule, on MXene and a metal-organic framework (MOF) and found that the adsorption of MB on MXene was better explained by the Freundlich model, while MB adsorption of MOFs was better explained by the Langmuir model [45]. In their work, MB adsorption on MXene was attributed to electrostatic interactions, which is in agreement with our findings through the determination of the Dubinin–Radushkevich model, while MB adsorption on MOF was attributed mainly to hydrophobic interactions. Similarly, in other studies, norfloxacin adsorption onto graphene oxide (GO) [46], carbon nanotubes (multi-walled CNTs) [47], and clays such as attapulgite-biochar [48] and zeolite [49] was also ascribed to hydrophobic interactions and π–π interactions. In these studies, they found that the adsorption of norfloxacin on these various adsorbents was explained best by the Langmuir isotherm model. The Langmuir fit in these studies implies that active adsorption is exclusively driven by interaction between the aromatic groups of norfloxacin and the amorphous carbon groups of the adsorbents. Interestingly, GO had an estimated norfloxacin adsorption capacity of 334.610 mg/g, while the MWCNTs had an absorption capacity of 87.0 mg/g. The adsorption of norfloxacin on clays such as attapulgite (modified with biochar) had a lower maximum adsorption capacity of 5.02 mg/g [48]. In our work, LiF/HCl-Ti_3_C_2_ nanosheets in suspension without the PVDF support layer (with an equivalent mass to MXene film of 0.25 mg MXene) had a comparable maximum adsorption capacity of 269.20 mg/g for norfloxacin to the referenced GO adsorbent investigation (see Table S18). Both GO and MXene have abundant surface groups, which could provide more adsorption active sites than a pristine and/or less functionalized carbon network of MWCNTs and other clay carbon-based adsorbents. This highlights that the surface functionality is an important feature for the high adsorption capacity of antibiotics in water as the surface groups can increase antibiotic uptake through dipole interactions. As MXene is composed mostly of titanium terminated with oxygen and fluorine groups, it is reasonable to expect that adsorption would be driven mainly by electrostatic interactions and hydrogen bonding rather than hydrophobic interactions. It should be noted that HF-Ti_3_C_2_ had some graphitic carbon due to over etching as exhibited in the XPS analysis, which could induce some extent of hydrophobic/π–π interactions that could contribute to the adsorption of antibiotics. Regardless, the antibiotics studied in this work are still relatively hydrophilic (partition coefficients Log D < 0.92), implying that the occurrence of hydrophobic interactions may be minimal. To further investigate the potential adsorption mechanism and the effect of electrostatic interactions and hydrogen bonding, an adsorption kinetic analysis and adsorption under different pH were conducted.

**Table 3:** Summary of the adsorption isotherm parameters from nonlinear fitting of the Freundlich and Dubinin–Radushkevich isotherms.

	Adsorption Isotherm Parameters				
		SMX	TMP	NOR	LEV
HF-Ti_3_C_2_	Freundlich				
	K_F_ [(mg/g)/(mg/L)^n^]	0.084	0.095	0.093	0.130
	n	0.880	0.874	0.915	1.011
	Adj. R^2^	0.957	0.959	0.986	0.916
	RMSE	0.088	0.103	0.049	0.126
	Dubinin–Radushkevich				
	Q_m_ [mg/g]	1.125	1.510	1.365	1.121
	E [KJ/mol]	0.440	0.389	0.357	0.532
	Adj. R^2^	0.947	0.935	0.920	0.798
	RMSE	0.097	0.130	0.115	0.195
HCl/HF-Ti_3_C_2_	Freundlich				
	K_F_ [(mg/g)/(mg/L)^n^]	0.081	0.161	0.346	0.147
	n	0.878	1.159	1.920	1.175
	Adj. R^2^	0.993	0.951	0.950	0.968
	RMSE	0.034	0.105	0.088	0.076
	Dubinin–Radushkevich				
	Q_m_ [mg/g]	1.406	1.217	1.058	1.131
	E [KJ/mol]	0.311	0.535	0.802	0.653
	Adj. R^2^	0.903	0.977	0.758	0.813
	RMSE	0.125	0.071	0.194	0.183
LiF/HCl-Ti_3_C_2_	Freundlich				
	K_F_ [(mg/g)/(mg/L)^n^]	0.100	0.197	0.498	0.228
	n	1.121	1.216	1.707	1.209
	Adj. R^2^	0.968	0.968	0.869	0.808
	RMSE	0.055	0.089	0.243	0.224
	Dubinin–Radushkevich				
	Q_m_ [mg/g]	0.760	1.281	1.412	1.199
	E [KJ/mol]	0.517	0.586	1.341	0.736
	Adj. R^2^	0.903	0.977	0.758	0.813
	RMSE	0.043	0.185	0.416	0.358
PVDF	Freundlich				
	K_F_ [(mg/g)/(mg/L)^n^]	3.35 × 10^−4^	0.103	0.006	0.023
	n	0.263	0.726	0.359	0.478
	Adj. R^2^	0.999	0.989	0.997	0.998
	RMSE	0.002	0.116	0.004	0.012
	Dubinin–Radushkevich				
	Q_m_ [mg/g]	5.503	3.389	0.877	3.951
	E [KJ/mol]	0.169	0.254	0.416	0.249
	Adj. R^2^	0.981	0.863	0.617	0.509
	RMSE	2.07 × 10^−4^	0.249	9.01 × 10^−5^	0.043

### 2.3. Adsorption Kinetic Modeling Analysis 

To gain a deeper understanding of the adsorption mechanisms of antibiotics on MXenes over time, kinetic model fitting was employed to analyze the results of the time-dependent batch adsorption experiments. All antibiotics reached equilibrium concentrations within approximately 90 min of the batch reaction, with the majority of the adsorption occurring within the first 30 min. Nonlinear pseudo-first order (PFO) and pseudo-second order (PSO) reaction kinetic models were used to describe the relationship between contact time and adsorption on active sites. Typically, a better fit of either PFO or PSO models to the experimental data (based on goodness-of-fit parameters) has been used to describe physisorption (PFO) or chemisorption (PSO) at the surface sites [50]. However, determining the main adsorption mechanism as chemisorption or physisorption based on reaction-based kinetic modeling is not always entirely conclusive [51]. Specifically, PFO has been found to be driven by the difference between saturation concentration and the amount of adsorbed mass at a given time, and therefore better describes adsorption that occurs in the initial stages of the process (i.e., the initial 20 to 30 min), rather than the entire process [50]. On the other hand, PSO has been found to be more suitable in describing kinetics throughout the entire adsorption process and describes adsorption that is driven by the specific sorption capacity of the adsorbent [52]. As each of the MXenes was composed of different surface chemistry and structures, their kinetics varied and imply that the adsorption kinetics of antibiotics on the MXenes is complex and affected by several factors. The parameters derived from fitting models for the time-dependent batch adsorption experiments are summarized in Table 4, Table S16, and Table S17. Based on the statistical parameters for each kinetics model (highest Adj. R^2^ and lowest RMSE), the reaction-based adsorption kinetics of SMX and TMP adsorption followed the PSO model on HF-Ti_3_C_2_ and the PFO model on HCl/HF-Ti_3_C_2_. On LiF/HCl-Ti_3_C_2_, SMX adsorption adhered to PSO kinetics, while TMP adsorption followed PFO kinetics. In the case of LEV and NOR, PSO kinetics described the adsorption over time better. Apart from a few cases where PFO was found to be the better model fit (i.e., SMX and TMP on HCl/HF-Ti_3_C_2_, TMP on LiF/HCl-Ti_3_C_2_), the PSO kinetic model fit better for most adsorption scenarios. According to the apparent reaction rate constants (k_2_, PSO fit) for LEV and NOR, the adsorption process occurred the fastest on PVDF and slowest on LiF/HCl-Ti_3_C_2_ (in order from fastest to slowest PVDF > HF-Ti_3_C_2_ > HCl/HF-Ti_3_C_2_ > LiF/HCl-Ti_3_C_2_). While adsorption was rapid with PVDF, the equilibrium concentration attained for each antibiotic was much lower than the equilibrium concentrations on LiF/HCl-Ti_3_C_2_ and other MXenes_._ This implies that there may be very few available sites for adsorption on PVDF, as evidenced by the very low adsorption capacities determined from the Freundlich isotherm fitting. This suggests that in the MXene–PVDF composite film, MXene plays a more significant role in adsorption.

In addition to reaction-based kinetic models, the intraparticle diffusion model (ID) was used to characterize the surface to pore diffusion. For the larger zwitterions of LEV and NOR, the intraparticle diffusion (ID) model fit the kinetic experiments better than PSO, particularly on LiF/HCl-Ti_3_C_2_. This implies that internal mass transfer through the MXene structure was the rate-limiting step in the overall adsorption process on LiF/HCl-Ti_3_C_2_. This could be attributed to the smaller interlayer spacing of LiF/HCl-Ti_3_C_2_ compared to other MXenes, as indicated by the XRD measurements (Table S1)_._ The size of these fluoroquinolone molecules might have hindered and restricted transport into and through the MXene pores and nanochannels, making it a significantly slower part of the process. The thickness boundary layer was represented by the intercept values, C (mg/g) (Table 4). For NOR on LiF/HCl-Ti_3_C_2,_ the impact of the boundary layer was relatively small in comparison to that of the other antibiotics. In the case of SMX and TMP, the ID fits were poor, and the large value of the boundary layer also supports the notion that intraparticle diffusion was not a dominant mechanism in the adsorption kinetics for those antibiotics. 

It is important to note that while kinetics fitting can help identify dominant kinetic steps at the solid–liquid interface, it does not entirely exclude the importance of other kinetic phenomena. For instance, the PSO and PFO kinetic models had very similar fits based on the determined Adj. R^2^ values for NOR and LEV on both LiF/HCl-Ti_3_C_2_ and HCl/HF-Ti_3_C_2_ (see Table 4). The slightly better fit of PSO for these antibiotics implies that surface chemisorption reactions are the primary mechanism for adsorption. However, this is contrary to the results of the Dubinin–Radushkevich model, which determined the adsorption of all MXene–antibiotic complexes to be driven by physisorption based on the derived adsorption energies (see Table 3). We can infer from these two seemingly different model determinations that the adsorption of the antibiotics onto MXene is complex. Chemical adsorption would imply the sharing of electrons in a covalent bond between the reactive functional groups of the antibiotics and surface groups of the MXenes [53]. On the other hand, physisorption involves weaker intermolecular interactions, amongst which hydrogen bonding is the strongest and is often a precursor step to form complexes [54,55]. Hydrogen bonding may be considered a special type of Lewis acid–Lewis base reaction [55] and is often a pivotal attractive force in the adsorption of pharmaceuticals [56,57]. This is a probable method of intermolecular interaction between the ample amount of hydrogen bond donors and acceptors of both the MXene surfaces and antibiotic moieties. Much of the adsorbed mass may be due to the MXene–antibiotic complex forged by hydrogen bonds. This may dually explain the suitability of PSO model kinetics and proximity of Adj. R^2^ values between the PSO and PFO kinetic models for many of the MXene–antibiotic adsorbed complexes, as hydrogen bonding is a strong attractive intermolecular force. This also indicates hydrogen donors (–OH terminations) and hydrogen acceptors (–O and –F terminations) as major specific antibiotic adsorption sites on the MXene surface. Overall, both isotherm and kinetic investigations indicate that LiF/HCl-Ti_3_C_2_ had the highest adsorption capacity. Besides internal mass transfer, surface interactions, likely hydrogen bonding, were the key drivers of adsorption. To further investigate this proposed mechanism, adsorption under different pH conditions and a closer examination of intermolecular binding could provide more insights into the impact of surface functionality on adsorption.

### 2.4. Effect of pH on Antibiotic Adsorption Capacity

**Figure 3:**
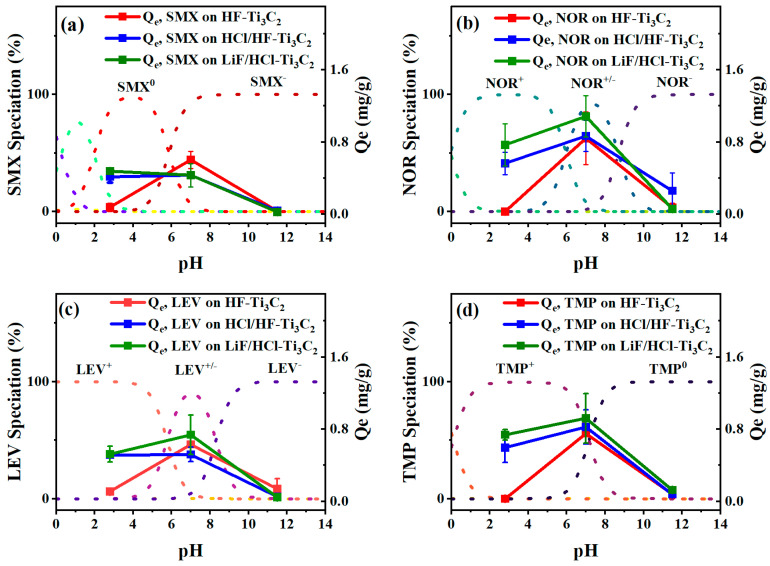
Adsorption capacities for (**a**) SMX, (**b**) NOR, (**c**) LEV, and (**d**) TMP at pH 2.8, pH 7, and pH 11.5 on HF-Ti_3_C_2_ (red), HCl/HF-Ti_3_C_2_ (blue), and LiF/HCl-Ti_3_C_2_ (green). Initial concentration C_0_ was 5 mg/L.

Given that both MXenes and antibiotic molecules possess chemical moieties that can be influenced by pH conditions, the effect of pH on the adsorption of the antibiotics (initial concentration, C_0_ = 5 mg/L) on the MXene membranes was systematically investigated under acidic conditions (pH 2.8), neutral conditions (pH 7), and basic conditions (pH 11.5). Zeta potential measurements (Figure S4) indicated that all MXenes retained a negative charge between pH 2 and 12, with the most pronounced negative charges (ζ < −57 mV) occurring above pH 9. On the other hand, the charges of the antibiotics displayed variations due to their ionizable functional groups, resulting in intriguing adsorption results, as depicted in Figure 3. 

As LiF/HCl-Ti_3_C_2_ and HCl/HF-Ti_3_C_2_ exhibited similar trends across the three pH conditions for each respective antibiotic, this discussion will focus on the differentiation between HF-Ti_3_C_2_ and LiF/HCl-Ti_3_C_2._ Notably, LiF/HCl-Ti_3_C_2_ consistently possessed a higher adsorption capacity. In the case of LiF/HCl-Ti_3_C_2_, SMX demonstrated its most effective adsorption under acidic conditions with an adsorbed mass of 0.479 mg/g, whereas it was 0.415 mg/g on HCl/HF-Ti_3_C_2_. This behavior could be attributed to the neutral charge SMX carried on the protonated amine within the oxazole group, facilitating electrostatic attraction between SMX and the slightly negatively charged MXene surface. However, under neutral and basic conditions, the amine group was deprotonated (with the strongest acidic pK_a_ = 5.86), increasing the negative charge on SMX and leading to electrostatic repulsion as the MXene surface became increasingly negative. This, in turn, resulted in reduced adsorption. Conversely, TMP carried a positive charge, which decreased under basic conditions (with the strongest basic pK_a_ = 7.16). TMP exhibited its highest adsorption at neutral pH (q_e_ on LiF/HCl-Ti_3_C_2_ = 0.920 mg/g, q_e_ on HCl/HF-Ti_3_C_2_ = 0.824 mg/g), followed by adsorption at acidic conditions and the lowest adsorption under basic conditions. At pH 2.8, TMP adsorption could be lower due to MXene becoming less negatively charged, thereby reducing the impact of electrostatic attraction between the positively charged TMP molecule and the MXene surface. At neutral conditions, TMP existed in two speciation forms with approximately 59% attributed to TMP^+^ and 41% attributed to TMP^0^, both of which could adsorb to the negatively charged MXene surface. However, TMP adsorption was the lowest at basic pH, likely due to TMP approaching a near-neutral charge, diminishing the electrostatic attraction with the negatively charged MXene surface. Additionally, TMP was the least hydrophilic of the antibiotics, as evident from distribution coefficient values summarized in Table S9, which further reduced the surface area contact between the molecule and surface under neutral and basic conditions. 

The highest adsorption levels were observed at neutral pH for LEV and NOR, with the overall highest adsorption result being NOR on LiF/HCl-Ti_3_C_2_ at neutral pH 7 (q_e_ = 1.08 mg/g). LEV and NOR are both zwitterions with similar moieties; at low pH, the carboxylic and amine groups were protonated, allowing for mild electrostatic attraction and reduced hydrogen bonding between the slightly negatively charged MXene surfaces and the cationic antibiotic molecules. The increased concentration of H^+^ ions at low pH could also compete with the cationic antibiotics, further reducing the adsorption of LEV and NOR in comparison to their adsorption performance at neutral pH, where a lower concentration of H^+^ ions would be present. These patterns of adsorption of antibiotics across pH conditions have been seen in previous works [47,58] and appear to be typical for zwitterionic molecules. At neutral pH, more NOR was adsorbed on all MXenes than LEV (0.842 mg NOR/g HF-Ti_3_C_2_, 0.632 mg LEV/g HF-Ti_3_C_2_; 0.868 mg NOR/g HCl/HF-Ti_3_C_2,_ 0.520 mg LEV/g HCl/HF-Ti_3_C_2_; 1.085 mg NOR/g LiF/HCl-Ti_3_C_2_, 0.738 mg LEV/g LiF/HCl-Ti_3_C_2_).This difference could be attributed to the higher percentage of LEV^ࢤ^ (ranging from 5.29 to 15.44%) compared to NOR^ࢤ^ (ranging from 8.36% to 22.62%), resulting in fewer LEV molecules being adsorbed by electrostatic attraction. Additionally, the positive amine group on LEV is bonded to a methyl group, which could hinder LEV’s attraction to the MXene surface. Under basic conditions, LEV and NOR were in anionic forms, leading to electrostatic repulsion with the MXene surface, as observed with all other antibiotics. However, antibiotic adsorption on HF-Ti_3_C_2_ was different from adsorption on HCl/HF-Ti_3_C_2_ and LiF/HCl-Ti_3_C_2_, with significantly lower adsorption under acidic conditions. This could be attributed to the competitive interaction of H^+^ ions with the highly electronegative -F surface groups, which may take precedence over interaction with the positively charged antibiotics in solution, resulting in low adsorption. Additionally, based on characterization of the material, a significant portion of the active film layer may not be composed of the MXene product (as indicated by Carbon XPS peak fitting). This could also reduce the charge–charge interaction, since many of these particles may be neutrally charged organics. However, under neutral conditions, HF-MXene remains competitive in comparison to the other two synthesized MXenes due to some extent of transient π–π electron–donor–acceptor (EDA) interactions between the aromatic rings in the antibiotics, charged moieties, and extraneous amorphous carbon. As the adsorption seems to be affected by pH conditions, this suggests that electrostatic interactions and hydrogen bonding are the dominant mechanisms facilitating the adsorption of the antibiotics. 

### 2.5. DFT Insight into Molecular Interactions between MXenes and Antibiotics

**Figure 4:**
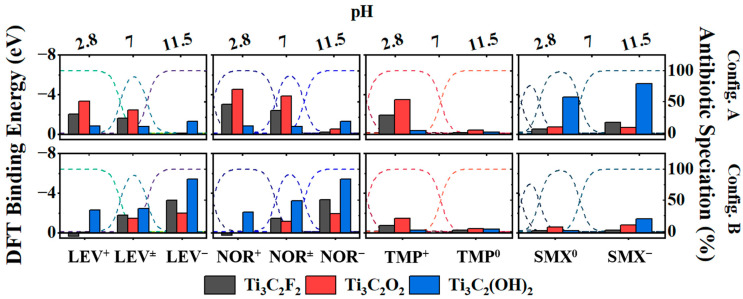
DFT binding energy calculation results of antibiotics to single termination MXene surfaces. Left-hand y-axis: DFT binding energy; right-hand y-axis: antibiotic speciation (%).

Given that the aforementioned experimental adsorption work suggests that intermolecular interactions may play an important role in adsorption, it is pertinent to delve into the intermolecular forces between antibiotic molecules and MXene surfaces to explain the superiority of LiF/HCl- and HCl/HF-derived MXenes in adsorption. Density functional theory (DFT) calculations were employed to unveil the effect of surface termination on adsorption behavior at an intermolecular level. A series of calculations was performed to simulate each antibiotic in the ionized states that occur within the experimental pH range of this work (pH 2.8–pH 11.5), positioned at a fixed proximity (2 Å) to single termination MXene surfaces with fluorine (Ti_3_C_2_F_2_), oxygen (Ti_3_C_2_O_2_), and hydroxyl (Ti_3_C_2_(OH)_2_) terminations. Different approach configurations (i.e., config. A and config. B) were used to simulate the interactions, considering that each antibiotic had distinct moieties that could interact with the MXene surface group, as displayed in Figure S6. The results of the DFT calculations are displayed in Figure 4 for each antibiotic speciation within the experimental pH ranges (LEV^+^, LEV^+/−^, LEV^−^ for LEV; NOR^+^, NOR^+/−^, NOR^-^ for NOR; TMP^+^ and TMP^0^ for TMP; SMX^0^ and SMX^−^ for SMX) on each type of MXene surface. The binding affinity was indicated by the binding energy values; the more negative the binding energy, the stronger the binding affinity between the MXene surface and the antibiotic. The fluoroquinolones, NOR and LEV, generally exhibited the strongest binding affinities, regardless of speciation and MXene surface termination. NOR, in particular, displayed the highest binding affinity, aligning with the experimental adsorption results. At neutral pH, the protonated amine (config. a) in both NOR and LEV displayed stronger binding affinities with Ti_3_C_2_O_2_ (NOR^+/−^ = −3.86 eV and LEV^+/−^ = −2.45 eV) and Ti_3_C_2_F_2_ (NOR^−^ = −3.39 eV and LEV^+/−^ = −1.60 eV), with the most substantial interaction occurring with the Ti_3_C_2_O_2_ surface. While both NOR^+/−^ and LEV^+/−^ carry protonated amine groups, the presence of the additional methyl group bonded to the nitrogen in LEV may have resulted in reduced charge–charge interaction between the -F/-O terminations of the MXenes and the protonated nitrogen of LEV, resulting in a lower binding energy. When the deprotonated carboxylic moieties (-COO^−^) of NOR^+/^ and LEV^+/−^ approached the MXene surface (config. b), they produced stronger binding energy values when in proximity to Ti_3_C_2_(OH)_2_ (NOR^+/−^ =−3.24 eV and LEV^+/−^ = −2.46 eV) than when in proximity to Ti_3_C_2_O_2_ (NOR^+/−^ =−1.17 eV and LEV^+/−^ = −1.45 eV) and Ti_3_C_2_F_2_ (NOR^+/−^ =−1.47 eV and LEV^+/−^ = −1.79 eV). This can be attributed to the deprotonated carboxylic group engaging in hydrogen bonding with the protons of the hydroxyl surface terminations. At neutral pH, TMP existed in two species, TMP^+^(58%) and TMP^0^ (41%), with the former showing high binding affinities due to the protonated nitrogen in the pyrimidine group (TMP^+^ on Ti_3_C_2_O_2_ = −3.51 eV, Ti_3_C_2_F_2_ = −1.91 eV, Ti_3_C_2_(OH)_2_ = −0.359 eV). The latter species, TMP^0^, exhibited a notably lower binding affinity. 

In the context of a pH environment, both hydrogen bonding and dispersion forces can significantly enhance and affect adsorption, as exhibited in the experimental work. Under acidic conditions, the dominant microspecies were positively charged (Z^+^ = NOR^+^, LEV^+^, and TMP^+^) and exhibited their strongest binding affinities with Ti_3_C_2_O_2_ and Ti_3_C_2_F_2_ (amine approach, config. A). However, it is important to note that the oxygen terminations of MXene are likely to be protonated under acidic conditions, given the excess amount of H^+^ at low pH, which would protonate existing –O– and –O^−^ terminations, leaving the available adsorption sites as –OH and –F terminations. Our DFT calculations indicated that the binding affinity between Z^+^ molecules and –OH and -F terminations was relatively low or even thermodynamically unfavorable (as exhibited in config. B with a binding energy greater than 0 eV when NOR^+^ and LEV^+^ approached Ti_3_C_2_F_2_), which aligned with our comparative adsorption experiments conducted under different pH conditions. Furthermore, while Ti_3_C_2_(OH)_2_ exhibited a notable binding affinity with the deprotonated carboxylic acid groups of the LEV^+/−^ and NOR^+/−^ antibiotics (config. B), the resulting affinities were still lower than those of amine-approached LEV^+/−^ and NOR^+/−^ to the Ti_3_C_2_O_2_ surface at neutral pH (config. A). This finding also supports why lower adsorption (as indicated by q_e_ values) of NOR, LEV, and TMP occurred at pH 2.8 (Z^+^) than at pH 7 (Z^+/−^), as the presence of the bare oxygen terminations (–O) would be higher at neutral pH than at low pH, under which negative oxygen groups become protonated (–OH). In contrast, SMX, while generally having a low binding affinity with –O, and –F terminations, showed a notably strong affinity for –OH terminations (config. A), likely due to hydrogen bonding and other van der Waals forces between the very electronegative sulfonamide group and the protons of the hydroxyls in Ti_3_C_2_(OH)_2_. Given the presence of protonated oxygen terminations (–OH) at low pH, this explained the slightly higher adsorption of SMX^0^ under acidic conditions than at neutral conditions (as seen in Figure 3). Under basic conditions when all antibiotics carry negative or neutral charges (LEV^−^, NOR^−^, SMX^−^, TMP^0^), the binding affinities were drastically smaller. Although these negatively charged antibiotics may have strong interactions with the –OH terminations based on the DFT calculations, under basic conditions, the MXene surface would predominantly consist of deprotonated terminations (–O^−^) due to the excess amount of hydroxyl ions in solution. This would result in a highly negative surface, as exhibited by the measured zeta potentials (Figure S4). Additionally, the DFT calculations also showed a very low binding affinity between all Z^−^ and Z^0^ antibiotics and MXene –F and –O terminations. From the DFT calculations, it can be determined that the presence of oxygen related terminations (both –O and –OH) drives adsorption due to electrostatic attraction. It is worth noting that while oxygen-terminations had the strongest binding affinities, the binding affinity of the antibiotics to fluorine-terminations was still quite significant, and these interactions played a role in adsorption at all pH conditions. 

While the DFT calculations could simulate the adsorption of antibiotics of single termination MXenes, it is important to acknowledge that MXenes often have mixed terminations, as evidenced from the characterization results. The DFT calculation highlighted that the –O and –OH terminations were the strongest binding sites for antibiotics, which may be why MXenes with a high oxygen content (such as LiF/HCl-Ti_3_C_2_ and HCl/HF-Ti_3_C_2_) exhibited a better adsorption performance. These calculations also revealed the key charged moieties of antibiotics that facilitate adsorption. In fact, molecular dynamic simulations conducted by Miri-Jahromi et al. revealed that the incorporation of functional groups onto Ti_2_C MXene increased the adsorption of penicillin molecules in comparison to adsorption onto bare Ti_2_C surfaces due to the increased electrostatic interactions [59]. Their study further elucidated that van der Waals interactions greatly contribute to the adsorption of the antibiotics as the aromatic and phenolic groups of the molecules could potentially serve as electron-rich sites for the hydroxyl groups of MXene at low and neutral pH conditions. In our work, both the DFT calculations and adsorption experimental work made it clear that the uptake of antibiotics was sensitive to pH conditions. This suggests that the application of MXene as an adsorbent film or membrane filter can be optimized based on pH to activate adsorption sites, specifically the activation of oxygen functional groups, for the selective removal of targeted pharmaceuticals. For instance, the DFT calculations revealed that SMX had its strongest binding energy to hydroxyl terminations due to hydrogen bonding. This could serve as a directive to utilize LiF/HCl-Ti_3_C_2_ MXene membranes at low pH for enhanced SMX removal. 

Antibiotic molecules that bypass molecular size selectively may be adsorbed to oxygen functional groups as they move through the composite membrane matrix. The size of the antibiotic may hinder penetration into the nanochannel and pores, given that some antibiotics may be larger than the MXene interlayer spacing (Figure S1). However, surface adsorption can still occur, and the surface chemistry derived from the synthesis conditions may affect the adsorption performance. While MXene membrane filtration primarily relies on size exclusion, this work implies that within the same antibiotic class, molecular size may not always be the sole determining factor for retention. For instance, in this work, it was demonstrated that the removal of NOR, despite its smaller size, may still have an edge over the removal of LEV within the same fluoroquinolone class due to their differing chemical moieties and receptive adsorption sites on MXenes, a phenomena that has been proposed and exhibited in previous investigations [11,60]. Our experimental findings in this work accentuate the importance of synthesis-derived surface chemistry as a key design feature to be optimized for pharmaceutical removal and other environmental applications.

## 3. Materials and Methods

### 3.1. Materials

The materials and reagents used in this study were as follows: commercial Ti_3_AlC_2_ (MAX) powder (Dingshengkeji Company, Shenzhen, China); hydrofluoric acid, 49–51 wt. % (Sigma-Aldrich, St. Louis, MO, USA); hydrochloric acid, 37 wt. % (ACS reagent, Sigma-Aldrich, St. Louis, MO, USA); sodium hydroxide, 20% (*w*/*w*) (RICCA chemical, Arlington, TX, USA); lithium fluoride, ≥98% (Alfa Aesar, Haverhill, MA, USA); lithium chloride, anhydrous, ≥98% (Alfa Aesar, Haverhill, MA, USA); formic acid, LC/MS Grade (Fisher Scientific, Hampton, NH, USA); polyvinylidene fluoride membrane filters, 0.2 micron, 47 mm (Sterlitech, Auburn, WA, USA); analytical standards of sulfamethoxazole, trimethoprim, levofloxacin, and norfloxacin were purchased from Sigma-Aldrich.

### 3.2. Preparation of Ti_3_C_2_T_x_ Nanosheets

#### 3.2.1. Preparation of HF-Ti_3_C_2_

HF-Ti_3_C_2_ was synthesized according to the protocol presented in a previous study [18]. Briefly, 1 g of commercial Ti_3_AlC_2_ powder was gradually added to 20 mL of 30 wt. % hydrofluoric acid in a high-density polyethylene (HDPE) container over a five-minute duration. The resulting mixture was stirred at 300 rpm using a magnetic stirrer for 5 h at room temperature. Subsequently, the mixture underwent thorough washing using 18.2 MΩ deionized (DI) water in several cycles of centrifugation at 3500 rpm for 10 min each, until the pH of the supernatant reached approximately pH 6. The resulting multi-layer Ti_3_C_2_T_x_ slurry was then delaminated by dispersing the slurry into 150 mL of DI water and subjecting the mixture to sonication under an argon atmosphere for 2 h. Delaminated HF-MXene was subsequently centrifuged at 3500 rpm for 1 h to remove any remaining unreacted MAX phase and stored for further processing.

#### 3.2.2. Preparation of HF/HCl-Ti_3_C_2_

HCl/HF-Ti_3_C_2_ was synthesized according to a previous study [61]. In a HDPE container, a solution was prepared by first adding 12 mL of hydrochloric acid (HCl) followed by the addition of 2 mL of hydrofluoric acid (HF) into 6 mL of DI water. Subsequently, 1 g of commercial Ti_3_AlC_2_ powder was carefully added over a five-minute duration into the acid mixture and left under continuous magnetic stirring at 300 rpm for 24 h. Following the reaction, the mixture was washed with 18.2 MΩ DI water involving several cycles of centrifugation at 3500 rpm for 10 min each until the pH of the supernatant reached around pH 6. The resulting multilayer MXene sediment was collected after concentration via centrifugation at 10,000 rpm for 10 min and dispersed in DI water to achieve a total volume of 20 mL in a container. To delaminate multilayer MXene material, 1 g of lithium chloride (LiCl) was added to the mixture. The mixture was then subjected to magnetic stirring at 300 rpm in a water bath maintained at 35 °C for 24 h. The subsequent removal of LiCl was accomplished through a series of centrifugation cycles, yielding a dark supernatant that was collected and stored for further processing. 

#### 3.2.3. Preparation of LiF/HCl-Ti_3_C_2_

LiF/HCl-Ti_3_C_2_ was synthesized based on the established ‘minimally intensive layer delamination’ or MILD method developed in a previous study [18]. In a HDPE bottle, 15 mL of 12 M HCl was added to 5 mL of 18.2 MΩ DI water to form a 20 mL solution of 9 M HCl. The mixture was stirred at 100 rpm for five minutes to ensure thorough mixing. Subsequently, 1.6 g of lithium fluoride (LiF) was dissolved into the acidic solution and stirred for 30 min to achieve homogeneity. Next, 1 g commercial Ti_3_AlC_2_ (MAX) powder was slowly added to the solution over a 5-min period. The HDPE bottle was securely placed in a water bath maintained at 35 °C and subjected to continuous magnetic stirring at 300 rpm for 24 h. The resulting mixture was washed through several cycles of centrifugation at 35,000 rpm for 10 min each, using 18.2 MΩ DI water until the pH of the supernatant approached approximately pH 6. The multilayer MXene suspension was subsequently sonicated under an argon atmosphere for 30 min. After sonication, the unreacted MAX phase sediment was effectively removed through centrifugation at 3500 rpm for 1 h, leaving behind the resultant MXene suspension, which was carefully stored for further processing.

#### 3.2.4. MXene Membrane Fabrication

MXene membranes were prepared by vacuum filtration of a MXene suspension onto 0.22 μm PVDF membrane filters. A specified quantity of MXene was dispersed in 100 mL of DI water and subjected to a 10-min sonication. The resulting suspension was then vacuum filtered onto a PVDF support and allowed to dry for 24 h at room temperature under vacuum conditions.

### 3.3. Characterization 

The synthesized MXenes were characterized comprehensively using a range of analytical techniques. Elemental composition and surface chemistry were assessed using X-ray photoelectron spectroscopy (XPS, Thermo K-Alpha XPS, Fisher Scientific, Hampton, NH, USA). To obtain morphological details and imagery, X-ray diffraction (XRD, Rigaku Miniflex Powder XRD, Tokyo, Japan) and a transmission electron microscope (TEM, JEOL 100 CX-II) were employed. Additionally, the zeta potential and lateral dimension of the MXenes was measured by a zeta potential and dynamic light scattering analyzer (Malvern Zetasizer Nano S, Worcestershire, UK).

### 3.4. Analytical Methodology

The quantification process involved both gradient and isocratic elution using an Agilent (1100 series) High Performance Liquid Chromatography-diode array detector (HPLC-DAD) equipped with a Zorbax C-18 column (3.0 × 150 mm, 3.5 um). The elution was carried out at a flow rate of 0.4 mL/min, utilizing a mobile phase A composed of 0.1% formic acid in 18.2 MΩ DI water (*v*/*v*), and mobile phase B consisting of methanol. The column temperature was maintained at 30 °C, and the sample injection volume was set to 25 µL. For detection purposes, the wavelengths were set at 270 nm for both sulfamethoxazole (SMX) and trimethoprim (TMP), 300 nm for levofloxacin (LEV), and 280 nm for norfloxacin (NOR). Prior to liquid chromatography analysis, all collected samples were collected and filtered through a 0.2 µm syringe filter. 

### 3.5. Adsorption Experiments 

Adsorption experiments were systematically conducted for each of the synthesized MXenes. In preparation for the adsorption experiments, the MXene-PVDF composite was adjusted to produce a MXene layer of 0.25 mg with an active area of 1.25 cm^2^ (area density: 0.21 mg/cm^2^) on a PVDF support (33.75 mg, area: 1.18 cm^2^). To initiate the adsorption isotherms, MXene film was added into a 50 mL centrifuge tube, along with varying initial concentrations of antibiotics. The concentrations of norfloxacin (NOR), trimethoprim (TMP), levofloxacin (LEV), and sulfamethoxazole (SMX) were adjusted to be a range from 0.5 to 10 mg/L including intermediate values of 1, 2, 3, 4, 5, and 7.5 mg/L. In the context of pH-dependent adsorption studies, the solutions were adjusted with HCl and NaOH to create three distinct pH conditions: low pH condition (pH 2.8), neutral pH condition (pH 7), and high pH condition (pH 11.5). The initial antibiotic concentration was consistently set at 5 mg/L for these experiments. All batch adsorption experiments were securely placed in a shaker and allowed to proceed for a duration of 24 h. The adsorption of antibiotics was quantified using the following equation:

$$q_{e}=\frac{\left(c_{0}-c_{e}\right)V}{m}$$

where $q_{e}$ (mg/g) represents the adsorption capacity at equilibrium (mg/g), V is the solution volume (L), and m denotes the mass of the adsorbent, which consists of both PVDF and MXene (g). Sample aliquots were collected before and after each experiment (initial and equilibrium concentrations, $c_{0}$ and $c_{e}$) and quantified using HPLC-DAD as described in the previous section. For the kinetic study, the adsorption of antibiotics was monitored at various time intervals within the same batch adsorption experiments previously described, spanning an 8-h duration. All batch adsorption experiments were conducted in triplicate. 

#### 3.5.1. Isotherm Equations

The Freundlich sorption isotherm model assumes adsorption on a heterogenous surface on sites with various energy of adsorption. The equation is as follows,

qe=KFCe1n

where $K_{F}$ is the Freundlich constant ((mg/g)(L/mg)^n^), $C_{e}$ (mg/L) denotes the equilibrium concentration of the adsorbate in the liquid phase, and *n* is an empirical factor that describes the availability and energy of adsorption of active sites [62].

The Dubinin–Radushkevich isotherm model can be expressed as

$$q_{e}=Q_{m}\exp(-\beta \varepsilon^{2})$$

where ε is the adsorption potential expressed as

$$\varepsilon =RTln(1+\frac{1}{C_{e}})$$

The sorption free energy (*E*, kJ/mol) can be calculated from β with the following equation:

$$E=\frac{1}{\sqrt[{2}]{2\beta }}$$

The details of the nonlinear and linear curve fitting, in addition to the statistical error analysis parameters used to assess the goodness-of-fit of the isotherms, are detailed in Supplementary Text S1.

#### 3.5.2. Kinetic Equations

The kinetic equations used in this study are shown in the equations below.

$$\mathrm{Pseudo}-\mathrm{first}\;\mathrm{order}\;(\mathrm{PFO}):\;q_{t}=q_{e} [1-\frac{1}{e^{k_{1}t}}]$$

$$\mathrm{Pseudo}-\mathrm{second}\;\mathrm{order}\;(\mathrm{PSO}):\;q_{t}=\frac{k_{2}q_{e}^{2}t}{1+k_{2}q_{e}t}$$

$$\mathrm{Intraparticle}\;\mathrm{diffusion}\;(\mathrm{ID}):\;q_{t}=k_{i}\sqrt[{2}]{t}+C$$

where $q_{t}$ (mg/g) is the adsorption capacity at time t (min), $q_{e}$ (mg/g) is the calculated adsorption capacity at equilibrium, $k_{1}$ (min^−1^), $k_{2}$ (g/mg-min), and $k_{i}$ (mg/g·min^−1/2^) are the kinetic rate parameters for the pseudo-first order, pseudo-second order, and intraparticle diffusion equations, respectively. 

### 3.6. Computational Calculations

The Vienna ab-initio simulation package (VASP) [63] was used to conduct the density functional theory (DFT) computational calculations, aiming to quantify binding energies of antibiotics to MXenes featuring various combinations of surface terminations. In these calculations, the Perdew–Burke–Ernzerhof (PBE) exchange-correlation functional within the framework of the generalized gradient approximation (GGA) was utilized. Periodic calculations were conducted with a plane-wave basis set using the projector-augmented-wave (PAW) method [63,64]. The DFT-D3 method was used to account for van der Waals and dispersion interactions [65]. To ensure accuracy, the MXene surfaces terminated with oxygen, fluorine, and hydroxyl groups were first optimized for energy and k-point convergence. A cut-off energy of 520 eV was identified and applied to larger slab-surface calculations. For binding energy calculations, a supercell (4 × 4) was created, containing 48 Ti-atoms, 32 C-atoms, and a total of 32 surface terminations (including single terminations of O, F, –OH) with a 15 Å vacuum gap to suppress the interaction between periodically repeated systems. In addition, a specific antibiotic was introduced at 2 Å above the MXene surface. Each MXene–antibiotic configuration underwent geometric relaxation until convergence was reached, with a criterion of 0.01 eVÅ^−1^ for ionic relaxation and 10^−6^ eV for the electronic self-consistency loop. Subsequently, single-point energy calculations were conducted without ionic relaxation to find the binding energy by using the following equation:

$$E_{B}=E_{AB+MX}-E_{AB}-E_{MX}$$

where *E_B_* (eV) represents the binding energy, *E_AB_*_+*MX*_ denotes the energy of the MXene–antibiotic complex, *E_AB_* is the energy of the antibiotic, and *E_MX_* represents the energy of the MXene surface.

## 4. Conclusions

Our findings shed light on the significant impact of the synthesis conditions on the resulting morphology and surface chemistry of MXenes. The results affirm the preference for mixed acid and bifluoride salt etching methods over the use of higher HF concentrated etchants, as these methods yield desirable MXene nanosheets suitable for adsorption and potential membrane filtration applications. The adsorption of antibiotics was found to vary based on the chemical structure of the molecule, with hydrogen bonding, van der Waals, and electrostatic interactions predominantly governing the binding of the antibiotics to the MXene surfaces. DFT calculations supported the experimental results of adsorption, revealing the favorable binding with oxygen terminations and, to a lesser extent, fluorine terminations. This highlights the possibility of tuning MXene surface chemistry to selectively remove certain antibiotics based on their characteristic moieties. Such insight can significantly improve the design, development, and application of MXene-based adsorption and membrane filtration, leveraging electrostatic interactions, and expanding the use of these membranes to achieve solute–solute separation for targeted emerging contaminant removal and resource recovery.

## Figures and Tables

**Table 1:** The XPS elemental composition is presented as atomic percentages (n.d. = not detected). These values represent an average derived from 4-layer depth profiling achieved through Ar^+^ sputtering, ensuring a global representation of the MXene’s overall composition.

XPS: Elemental Composition (Atom %)				
	HF-Ti_3_C_2_	HCl/HF-Ti_3_C_2_	LiF/HCl-Ti_3_C_2_	PVDF
**Ti**	19.40	34.77	33.14	n.d.
**C**	41.77	31.96	29.76	77.58
**O**	12.99	20.57	20.99	0.66
**F**	23.87	7.67	11.76	21.76
**Cl**	n.d.	1.61	2.72	n.d.

**Table 4:** Summary of the adsorption kinetics fitting and parameters. Initial concentration (C_0_) of 1 mg/L.

	Adsorption Kinetic Parameters				
		SMX	TMP	NOR	LEV
HF-Ti_3_C_2_	PFO				
	k_1_ [min^−1^]	0.133	14.281	0.041	0.109
	Q_e_ [mg/g]	0.011	0.056	0.142	0.063
	Adj. R^2^	0.599	−1.550	0.593	0.747
	RMSE	0.003	0.034	0.045	0.014
	PSO				
	k_2_ [g/mg·min^−1^]	14.725	2.29 × 10^2^	0.205	2.392
	Q_e_ [mg/g]	0.011	0.045	0.172	0.068
	Adj. R^2^	0.638	0.696	0.657	0.776
	RMSE	0.003	0.012	0.042	0.013
	ID				
	k_i_ [mg/g·min^−1/2^]	0.001	0.001	0.010	0.004
	C [mg/g]	0.003	0.028	0.015	0.019
	Adj. R^2^	0.631	−0.075	0.736	0.608
	RMSE	0.003	0.022	0.002	0.018
HCl/HF-Ti_3_C_2_	PFO				
	k_1_ [min^−1^]	0.042	0.039	0.006	0.065
	Q_e_ [mg/g]	0.024	0.061	0.125	0.040
	Adj. R^2^	0.808	0.787	0.637	0.769
	RMSE	0.005	0.012	0.023	0.009
	PSO				
	k_2_ [g/mg·min^−1^]	1.984	0.969	0.129	1.772
	Q_e_ [mg/g]	0.026	0.066	0.112	0.044
	Adj. R^2^	0.737	0.760	0.676	0.769
	RMSE	0.009	0.019	0.017	0.011
	ID				
	k_i_ [mg/g·min^−1/2^]	0.001	0.003	0.006	0.003
	C [mg/g]	0.005	0.015	0.005	0.009
	Adj. R^2^	0.426	0.490	0.796	0.648
	RMSE	0.009	0.019	0.017	0.011
LiF/HCl-Ti_3_C_2_	PFO				
	k_1_ [min^−1^]	0.055	0.033	0.006	0.007
	Q_e_ [mg/g]	0.022	0.156	0.527	0.339
	Adj. R^2^	0.815	0.992	0.890	0.960
	RMSE	0.004	0.006	0.054	0.022
	PSO				
	k_2_ [g/mg·min^−1^]	4.158	0.236	0.008	0.011
	Q_e_ [mg/g]	0.023	0.979	0.703	0.480
	Adj. R^2^	0.840	0.973	0.899	0.964
	RMSE	0.004	0.011	0.051	0.021
	ID				
	k_i_ [mg/g·min^−1/2^]	0.001	0.010	0.025	0.018
	C [mg/g]	0.006	0.024	0.000	−0.009
	Adj. R^2^	0.610	0.794	0.951	0.975
	RMSE	0.006	0.030	0.036	0.018
PVDF	PFO				
	k_1_ [min^−1^]	0.017	0.013	0.175	0.068
	Q_e_ [mg/g]	0.001	0.076	0.107	0.027
	Adj. R^2^	0.927	0.781	0.995	0.867
	RMSE	1.46 × 10^−4^	0.014	0.003	0.004
	PSO				
	k_2_ [g/mg·min^−1^]	7.982	0.214	3.898	3.322
	Q_e_ [mg/g]	0.002	0.086	0.110	0.029
	Adj. R^2^	0.920	0.833	0.996	0.895
	RMSE	1.53 × 10^−4^	0.012	0.003	0.004
	ID				
	k_i_ [mg/g·min^−1/2^]	1.00 × 10^−4^	0.005	0.005	0.002
	C [mg/g]	−1.00 × 10^−4^	0.005	0.046	0.007
	Adj. R^2^	0.878	0.928	0.425	0.722
	RMSE	1.89 × 10^−4^	0.008	0.033	0.006

## Data Availability

The data presented in this study are available on request from the corresponding author.

## Supplementary Materials

The following supporting information can be downloaded at: https://www.mdpi.com/article/10.3390/molecules29010148/s1, Figure S1: XPS survey depth profile; Figure S2: XPS peak-fit spectrums with no etching; Figure S3: XPS depth profile of the Ti 2p region; Figure S4: Zeta potential of MXenes; Figure S5: Adsorption isotherms; Figure S6: Schematic of antibiotic (configurations A and B) on MXene surfaces; Table S1: XRD of dry and wet MXene films; Tables S2–S8: XPS peak fitting details inclusive of binding energy position, full width at half maximum values (FWHM), atomic composition, and assignments; Table S9: Distribution coefficients of antibiotics; Tables S1–S15: Freundlich, Langmuir, and Dubinin–Radushkevich adsorption; Tables S16–S17: PFO and PSO kinetic fitting. Table S18: Summary of comparative adsorption isotherm and kinetic studies. References [66,67,68,69] are cited in the Supplementary Materials.
