# Supplementary material for: The Impact of Surface Chemistry and Synthesis Conditions on the Adsorption of Antibiotics onto MXene Membranes
Source: Molecules. 2023 Dec 26;29(1):148. doi: 10.3390/molecules29010148 (PMC10780216; doi:10.3390/molecules29010148)
Supplement: Supplementary file 1 [file molecules-29-00148-s001.zip › molecules-2721162-supplementary.pdf]

SUPPLEMENTARY MATERIALS

The Impact of Surface Chemistry and Synthesis Conditions on the Adsorption of Antibiotics onto MXene Membranes

Moyosore A. Afolabi [1], Dequan Xiao [2] and Yongsheng Chen [1,]*

[1] School of Civil and Environmental Engineering, Georgia Institute of Technology, Atlanta, GA 30332, USA

[2] Center for Integrative Materials Discovery, Department of Chemistry and Chemical & Biomedical Engineering, University of New Haven, West Haven, CT 06516, USA

* Correspondence: yongsheng.chen@ce.gatech.edu

Number of pages: 32

Number of texts: 1

Number of figures: 7

Number of tables: 18

X-Ray Diffraction (XRD)

MXene Films	Bragg Angle 2*θ*(°)		d-spacing (Å)		Interlayer Spacing (Å)
	Dry Film	Wet Film	Dry Film	Wet Film	
HF-Ti_3C_2	6.496	5.244	13.596	16.830	3.234
HCl/HF-Ti_3C_2	6.516	5.050	13.554	17.475	3.921
LiF/HCl-Ti_3C_2	6.261	5.556	14.105	15.888	1.783

Table S1. XRD on MXene films: Bragg angle and calculated d-spacing of (002) peak.

X-Ray Photoelectron Spectra (XPS) Analysis

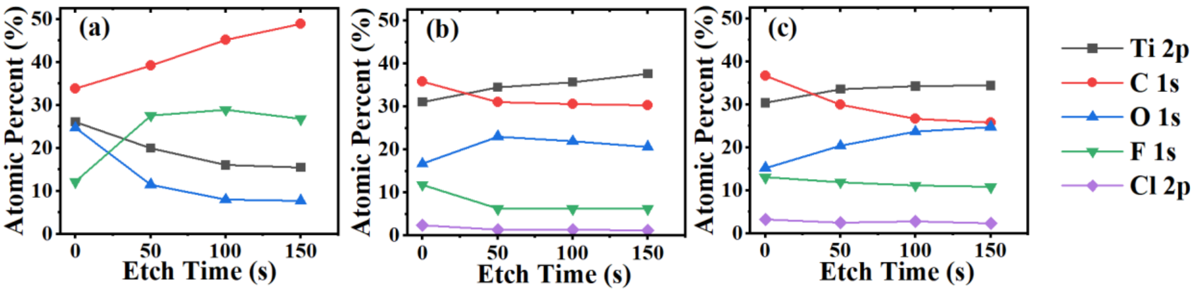

Figure S1.XPS depth profile (Ar^+ sputtering) of MXenes. (**a**) HF-Ti_3C_2, (**b**) HCl/HF-Ti_3C_2, and (**c**) LiF/HCl-Ti_3C_2. Etch time 0 seconds = etch level 0, Etch time 50 seconds = etch level 1.

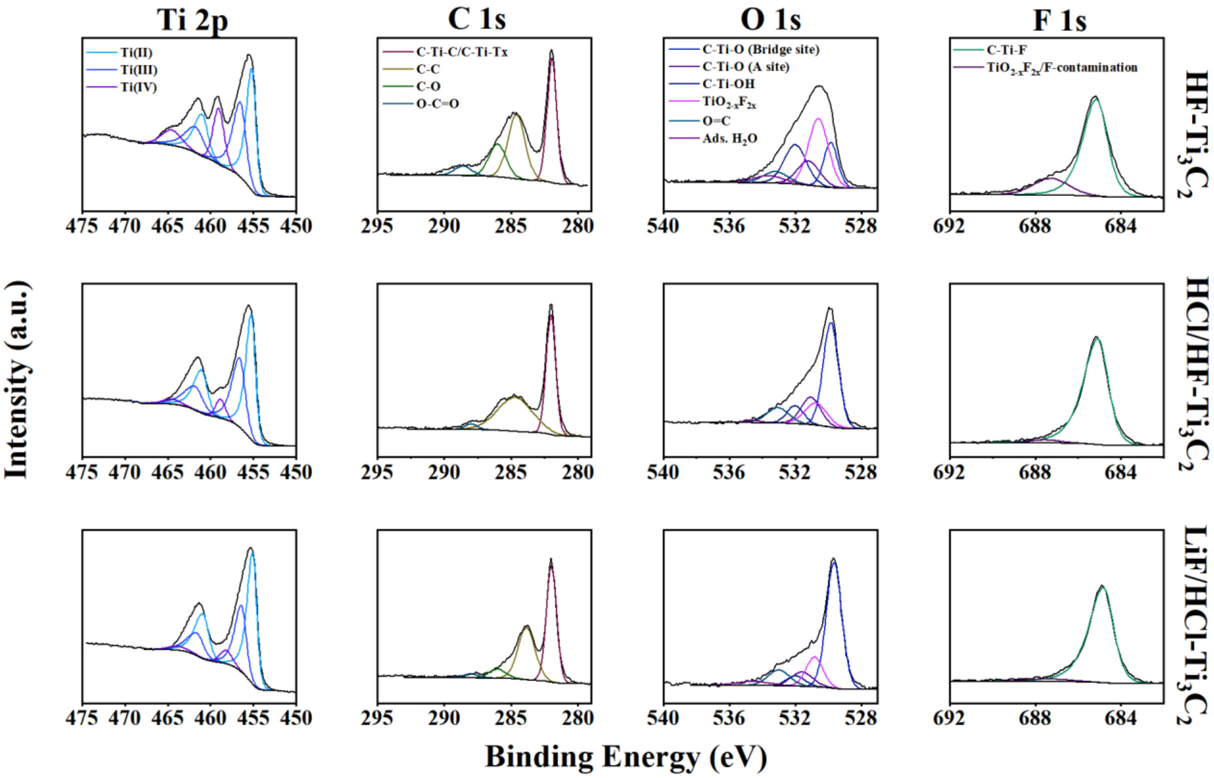

Figure S2. XPS peak-fit narrow scan regions with no Ar^+ (etch time 0 s) for ($HF-Ti_3C_2$) (**a**–**d**), ($HCl/HF-Ti_3C_2$) (**e**–**h**), and ($LiF/HCl-Ti_3C_2$) (**i**–**l**).

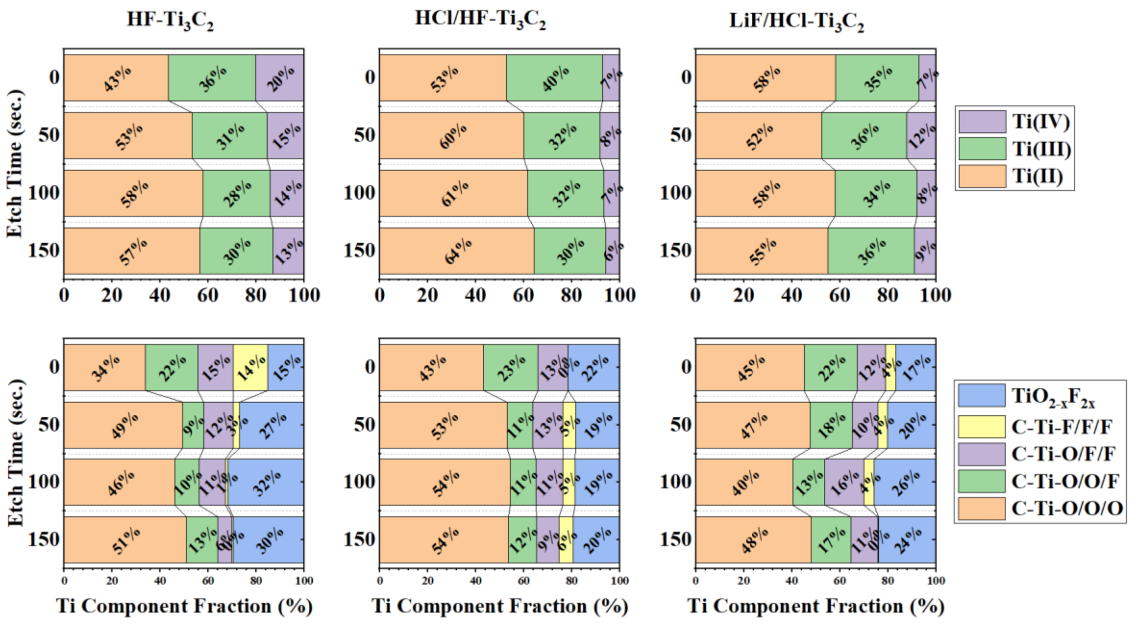

Figure S3. Depth profile of Ti 2p narrow scan region of MXenes. Peak fitting with Ti oxidation in the first row and peak fitting scheme by chemical environment in the bottom row.

Table S2. XPS peak fitting of HF-Ti_3C_2 without Ar^+ sputtering/depth profiling (etch level 0, 0 s etching). MXene components are indicated under the assignment column. FWHM = full width at half maximum.

Region	BE (eV)	FWHM (eV)	At. %	Assignment
Ti $2p_{3/2}$ Ti $2p_{1/2}$	455.18	1.1	11.17	Ti^{2+} (MXene product)
	460.98	1.6		
	456.51	1.5	9.35	Ti^{3+} (MXene product)
	461.71	2.2		
	459.07	1.5	5.27	Ti^{4+}
	464.57	2.7		
C 1s	281.96	0.9	15.70	C-Ti-C/C-Ti-T_x (MXene product)
	284.60	1.5	10.84	C-C
	286.02	1.5	5.57	C-O
	288.60	1.5	1.64	O-C=O
O 1s	529.85	1.07	4.36	C-Ti-O (bridge site) (MXene product)
	531.20	1.4	3.76	C-Ti-O (A site) (MXene product)
	533.20	1.2	6.04	C-Ti-OH (MXene product)
	530.60	1.2	9.25	$TiO_{2-x}F_{2x}$
	533.20	3.6	2.02	O=C
	533.60	2.0	1.54	Ads. H_2O
F 1s	685.07	1.3	10.90	C-Ti-F (MXene product)
	687.55	2.0	2.59	$TiO_{2-x}F_{2x}$/F-contamination

Table S3. XPS peak fitting of HF-Ti_3C_2 with Ar^+ sputtering/depth profiling (etch level 1, 50 s etching). MXene components are indicated under the assignment column.

Region	BE (eV)	FWHM (eV)	At. %	Assignment
Ti $2p_{3/2}$ Ti $2p_{1/2}$	454.86	1.1	9.53	Ti^{2+}
	460.66	1.6		(MXene product)
	456.50	1.5	5.63	Ti^{3+}
	461.70	2.2		(MXene product)
	459.02	1.7	2.77	Ti^{4+}
	464.72	3.2		
C 1s	281.91	0.9	7.21	C-Ti-C/C-Ti-T_x
				(MXene product)
	282.80		4.20	C-Ti-O
		1.4		(MXene product)
	284.60	1.5	18.27	C-C
	286.10	1.5	7.57	C-O
	288.20	1.4	2.14	O-C=O
	290.15	1.4	4.15	C=O
O 1s	529.89	1.07	1.59	C-Ti-O (bridge site)
				(MXene product)
	531.00	1.4	4.93	C-Ti-O (A site)
				(MXene product)
	531.93	1.2	1.58	C-Ti-OH
				(MXene product)
	530.61	1.2	2.12	$TiO_{2-x}F_{2x}$
	533.22	3.6	1.17	O=C
	533.72	2.0	0.17	Ads. H_2O
F 1s	685.2	1.3	12.58	C-Ti-F
				(MXene product)
	686.9	2.0	17.82	$TiO_{2-x}F_{2x}$/F- contamination

Table S4. XPS peak fitting of HCl/HF-Ti_3C_2 with Ar^+ sputtering/depth profiling (etch level 0, 0 s etching). MXene components are indicated under the assignment column.

Region	BE (eV)	FWHM (eV)	At. %	Assignment
Ti $2p_{3/2}$<br>Ti $2p_{1/2}$	455.23<br>461.03	1.1<br>1.6	15.94	Ti^{2+}<br>(MXene product)
	456.60<br>461.80	1.5<br>2.2	12.01	Ti^{3+}<br>(MXene product)
	458.84<br>464.54	1.3<br>2.5	2.23	Ti^{4+}
C1s	282.03	0.8	19.69	C-Ti-C/C-Ti-T_x<br>(MXene product)
	284.71	3.06	20.57	C-C
	287.98	1.1	1.19	C=O
O1s	529.84	1.0	5.93	C-Ti-O (bridge site)<br>(MXene product)
	531.06	1.6	2.02	C-Ti-O (A site)<br>(MXene product)
	532.00	1.6	1.13	C-Ti-OH<br>(MXene product)
	530.75	1.6	1.86	$TiO_{2-x}F_{2x}$
	533.06	1.5	1.29	O=C
	534.60	1.9	0.13	Ads. H_2O
F 1s	685.07	1.2	13.24	C-Ti-F<br>(MXene product)
	6867.55	1.7	0.44	$TiO_{2-x}F_{2x}$/F-contamination
Cl 2p	200.08	-	2.31	C-Ti-Cl<br>(MXene product)

Table S5. XPS peak fitting of HCl/HF-Ti_3C_2 with Ar^+ sputtering/depth profiling (etch level 1, 50 s etching). MXene components are indicated under the assignment column.

Region	BE (eV)	FWHM (eV)	At. %	Assignment
Ti $2p_{3/2}$ Ti $2p_{1/2}$	454.89	1.1	20.44	Ti^{2+} (MXene product)
	460.69	1.6		
	456.69	1.5	10.92	Ti^{3+} (MXene product)
	461.70	2.2		
	458.50	1.6	2.82	Ti^{4+}
	464.20	3.0		
C1s	281.94	0.8	9.24	C-Ti-C/C-Ti-T_x (MXene product)
	282.42	2.0	10.47	C-Ti-O (MXene product)
	284.28	1.5	3.48	C-C
	286.29	2.7	10.11	C-O
	289.08	1.1	0.93	C=O
O1s	529.5	1.0	0.38	C-Ti-O (bridge site) (MXene product)
	531.01	1.6	12.02	C-Ti-O (A site) (MXene product)
	532.00	1.6	3.17	C-Ti-OH (MXene product)
	530.20	1.6	6.21	$TiO_{2-x}F_{2x}$
	533.00	1.5	2.28	O=C
	534.60	1.9	0.88	Ads. H_2O
F 1s	684.99	1.2	4.66	C-Ti-F (MXene product)
	686.80	1.7	1.16	$TiO_{2-x}F_{2x}$/F-contamination

Cl 2p	199.91	-	0.79	C-Ti-Cl (MXene product)

Table S6. XPS peak fitting of LiF/HCl-Ti_3C_2 with Ar^+ sputtering/depth profiling (etch level 0, 0 s etching). MXene components are indicated under the assignment column.

Region	BE (eV)	FWHM (eV)	At. %	Assignment
Ti $2p_{3/2}$<br>Ti $2p_{1/2}$	455.08	1.1	15.91	Ti^{2+}
	460.88	1.6		(MXene product)
	456.40	1.3	9.50	Ti^{3+}
	461.60	1.9		(MXene product)
	458.20	1.7	1.92	Ti^{4+}
	463.80	3.0		
C1s	282.99	0.9	22.82	C-Ti-C/C-Ti-T_x<br>(MXene product)
	283.85	1.5	15.97	C-C
	284.88	1.5	3.73	C-O
	286.01	1.37	1.16	C=O
O1s	529.65	1.0	7.68	C-Ti-O (bridge site)<br>(MXene product)
	531.60	1.6	1.13	C-Ti-O (A site)<br>(MXene product)
	531.95	1.6	0.69	C-Ti-OH<br>(MXene product)
	530.84	1.6	2.00	$TiO_{2-x}F_{2x}$
	533.00	1.5	1.42	O=C
	534.55	1.9	0.19	Ads. H_2O
F 1s	684.82	1.2	12.05	C-Ti-F<br>(MXene product)
	687.40	1.7	0.56	$TiO_{2-x}F_{2x}$/F-<br>contamination
Cl 2p	200.51		3.13	C-Ti-Cl<br>(MXene product)

Table S7. XPS peak fitting of LiF/HCl-Ti_3C_2 with Ar^+ sputtering/depth profiling (etch level 1, 50 s etching). MXene components are indicated under the assignment column.

Region	BE (eV)	FWHM (eV)	At. %	Assignment
Ti $2p_{3/2}$ Ti $2p_{1/2}$	454.77	1.1	18.08	Ti^{2+}
	460.47	1.6		(MXene product)
	456.13	1.5	12.30	Ti^{3+}
	461.33	2.2		(MXene product)
	458.03	1.7	4.19	Ti^{4+}
	463.72	3.0		
C1s	281.94	0.8	8.94	C-Ti-C/C-Ti-T_x (MXene product)
	282.51	2.0	12.86	C-Ti-O (MXene product)
	284.57	1.5	6.22	C-C
	286.49	1.5	2.37	C-O
	288.5	2.1	1.21	O-C=O
O1s	529.74	1.0	2.92	C-Ti-O (bridge site) (MXene product)
	531.20	1.6	4.63	C-Ti-O (A site) (MXene product)
	532.00	1.6	2.17	C-Ti-OH (MXene product)
	530.51	1.6	6.22	$TiO_{2-x}F_{2x}$
	533.00	1.5	1.57	O=C
	534.60	1.9	0.88	Ads. H_2O
F 1s	684.88	1.2	10.11	C-Ti-F (MXene product)
	686.80	1.7	1.20	$TiO_{2-x}F_{2x}$/F-contamination

Cl 2p	200.52		3.16	C-Ti-Cl<br>(MXene product)

Table S8. XPS peak fitting of MXenes. Values are an average of the peak fitted values over the 4-layer depth profile.

	Region	BE (eV)	FWHM (eV)	Fraction	Assignment
**HF-Ti_3C_2**	Ti $2p_{3/2}$	455.0	1.3	0.45	C-Ti-O/O/O
	Ti $2p_{1/2}$	461.1	1.5		(MXene product)
		456.4	1.3	0.14	C-Ti-O/O/F
		462.5	1.5		(MXene product)
		457.1	1.3	0.11	C-Ti-O/F/F
		463.2	1.5		(MXene product)
		459.4	1.3	0.05	C-Ti-F/F/F
		465.1	1.5		(MXene product)
		459.4	2.4	0.26	$TiO_{2-x}F_{2x}$
		465.1	3.2		
**HCl/HF-Ti_3C_2**	Ti $2p_{3/2}$	455.0	1.3	0.51	C-Ti-O/O/O
	Ti $2p_{1/2}$	461.1	1.6		(MXene product)
		456.3	1.3	0.14	C-Ti-O/O/F
		462.4	1.6		(MXene product)
		457.0	1.3	0.12	C-Ti-O/F/F
		463.1	1.6		(MXene product)
		457.9	1.3	0.04	C-Ti-F/F/F
		464.0	1.6		(MXene product)
		459.5	2.3	0.20	$TiO_{2-x}F_{2x}$
		465.1	3.3		
**LiF/HCl-Ti_3C_2**	Ti $2p_{3/2}$	455.1	1.3	0.45	C-Ti-O/O/O
	Ti $2p_{1/2}$	461.1	1.4		(MXene product)
		456.1	1.3	0.17	C-Ti-O/O/F
		462.2	1.4		(MXene product)
		457.1	1.3	0.13	C-Ti-O/F/F
		463.2	1.4		(MXene product)

	457.9	1.3	0.02	C-Ti-F/F/F
	464.0	1.4		(MXene product)
	459.4	2.6	0.22	$TiO_{2-x}F_{2x}$
	465.0	3.3		

Zeta Potential Analysis

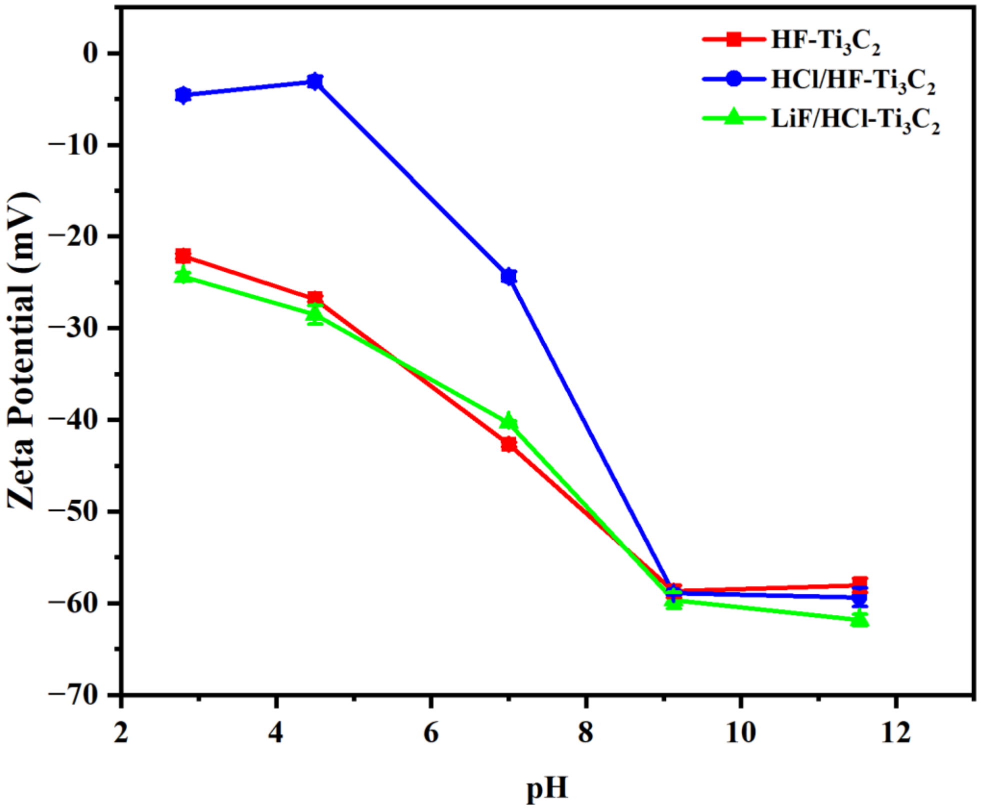

Figure S4. Zeta potential of MXene suspension at pH 2.8 to pH 11.5.

Table S9. Distribution coefficients of ionizable antibiotics at different pH. Data sourced from ChemAxon's Chemcalize[1].

**Antibiotic**	**Log D at pH 2.8**	**Log D at pH 7**	**Log D at pH 11.5**
SMX	0.73	0.03	−0.15
TMP	−0.19	0.92	1.28
NOR	−1.80	−0.87	−2.07
LEV	−1.93	−0.80	−1.97

Text S1. Adsorption Experimental Data, Regression, and Statistical Analysis

All regression (linear and nonlinear) analysis was conducted in OriginPro, Version 2023b (OriginLab Corporation). All nonlinear curve fitting was conducted with the Levenberg–Marquardt minimization algorithm (damped least-squares), with iterations conducted to a tolerance of χ^2 = 1E-9 [2]. The nonlinear and linear isotherm equations are displayed below.

Freundlich	$q_e = K_F C_e^{1/n}$	$\frac{1}{q_e} = \left(\frac{1}{K_L q_m}\right)\frac{1}{C_e} + \frac{1}{q_m}$
Langmuir	$\frac{K_L q_m C_e}{1 + K_L C_e}$	$\frac{1}{q_e} = \left(\frac{1}{K_L q_m}\right)\frac{1}{C_e} + \frac{1}{q_m}$
Dubinin–Radushkevich	$q_e = q_m \exp(-\beta\varepsilon^2)$	$\ln q_e = \ln q_m - \beta\varepsilon^2$ $\varepsilon = RT \ln\left(1 + \frac{1}{C_e}\right)$

Statistical error parameters of the coefficient of determination (R^2). The adjusted R^2, reduced chi-square (Red. χ^2), sum of squared error or residual sum of squares (SSE or RSS), and the root mean square error (RMSE) were calculated within the OriginPro software and used to assess the 'goodness-of-fit' of each of the isotherm models for the antibiotic adsorption experiments.

The Dubinin–Radushkevich and Freundlich isotherm details are outlined within the main manuscript in the Materials and Methods section. Langmuir is an appropriate fit for antibiotic adsorption (regardless of linear or nonlinear fitting), however, details of the isotherm are described below.

Additional isotherm fitting: Langmuir isotherm

The Langmuir sorption isotherm model assumes monolayer adsorption on a homogenous surface with equally available and energetic sites. The equation is as follows:

Langmuir: $$q_e = \frac{q_m K_L C_e}{1 + K_L C_e}$$

where K_L (L/mg) is the Langmuir constant and q_m (mg/g) is the maximum adsorption capacity. The separation factor, R_L, is a dimensionless number that describes the favorability of the adsorption process, and the equation is as follows,

$$R_L = \frac{1}{1 + K_L C_0}$$

where C_0 (mg/L) is the initial concentration of adsorbate. $0 < R_L < 1$ denotes favorable adsorption, while $R_L > 1$ denotes unfavorable adsorption, $R_L = 0$ indicates irreversible adsorption and $R_L = 1$ indicates that the adsorption isotherm follows a linear profile.

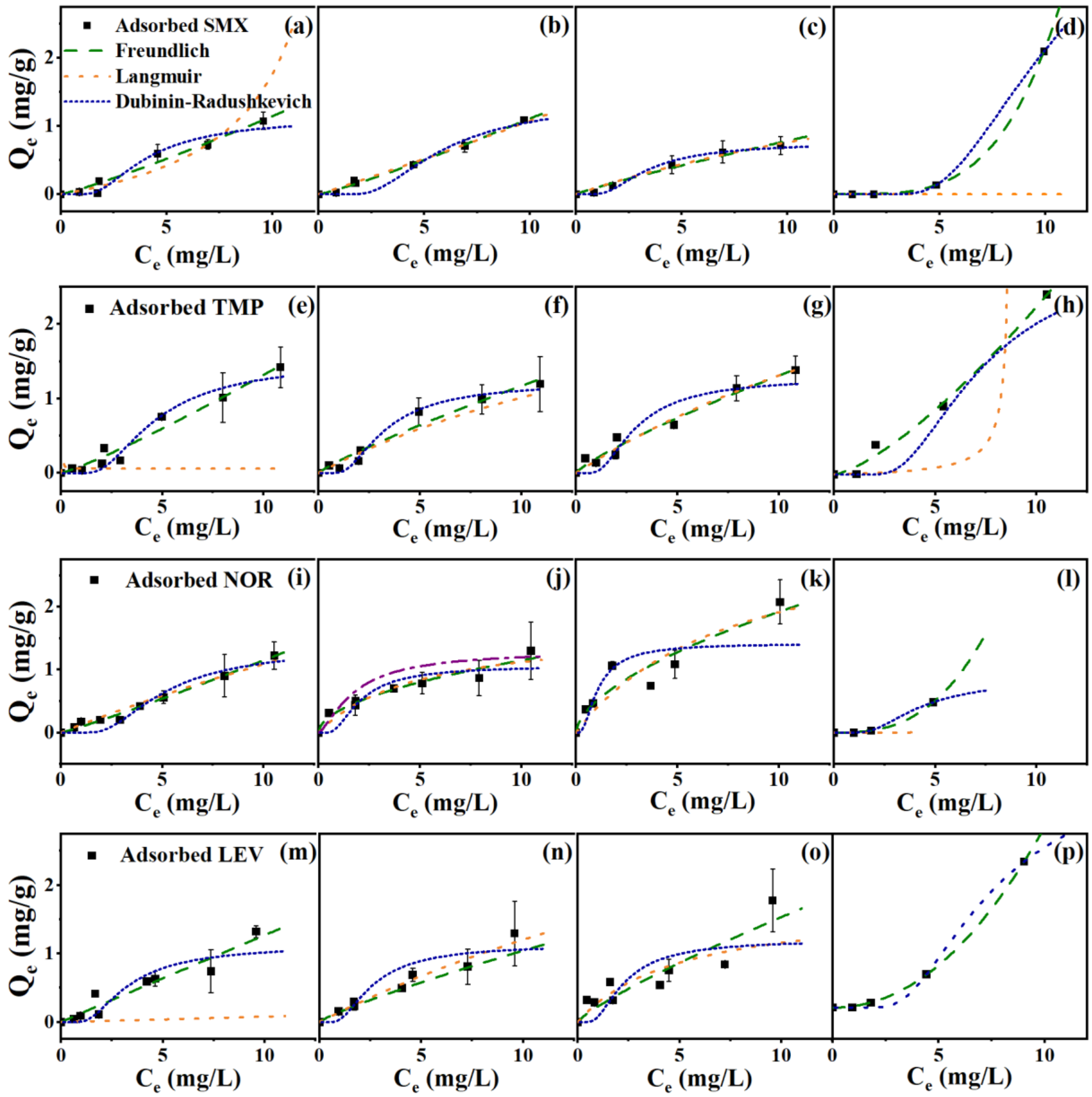

Figure S5. Adsorption isotherm experimental data with the Freundlich (green), Langmuir (orange), and Dubinin–Radushkevich (blue) nonlinear fits for SMX, TMP, NOR, and LEV. Adsorption of antibiotic on HF-Ti3C2 (**a**,**c**,**i**,**m**), HCl/HF-Ti3C2 (**b**,**f**,**j**,**n**), LiF/HCl-Ti3C2 (**c**,**g**,**k**,**o**), and PVDF (**d**,**h**,**l**,**p**).

Table S10. Summary of the Freundlich adsorption isotherm fitting parameters and statistical error analysis from nonlinear regression.

		Freundlich Nonlinear Fitting						
		**K_F [(mg/g)/(mg/L)^n]**	**n**	**SSE**	**R^2**	**Adj. R^2**	**Red. χ^2**	**RMSE**
**HF-Ti_3C_2**	**SMX**	0.084	0.880	0.039	0.964	0.957	0.008	0.088
	**TMP**	0.095	0.874	0.074	0.964	0.959	0.011	0.103
	**NOR**	0.093	0.915	0.017	0.987	0.986	0.002	0.049
	**LEV**	0.130	1.011	0.111	0.926	0.916	0.016	0.126
**HCl/HF-Ti_3C_2**	**SMX**	0.081	0.878	0.006	0.994	0.993	0.001	0.034
	**TMP**	0.161	1.159	0.066	0.958	0.951	0.011	0.105
	**NOR**	0.346	1.920	0.046	0.958	0.950	0.008	0.088
	**LEV**	0.147	1.069	0.035	0.972	0.968	0.006	0.076
**LiF/HCl-Ti_3C_2**	**SMX**	0.100	1.121	0.012	0.975	0.968	0.003	0.055
	**TMP**	0.197	1.216	0.047	0.973	0.968	0.008	0.089
	**NOR**	0.498	1.707	0.296	0.891	0.869	0.059	0.243
	**LEV**	0.228	1.209	0.350	0.832	0.808	0.050	0.224
**PVDF**	**SMX**	0.000	0.263	0.000	0.999	1.000	0.000	0.002
	**TMP**	0.103	0.726	0.040	0.992	0.989	0.013	0.116
	**NOR**	0.006	0.359	0.000	1.000	1.000	0.000	0.004
	**LEV**	0.023	0.478	0.000	0.999	1.000	0.000	0.012

Table S11. Summary of the Freundlich adsorption isotherm fitting parameters and statistical error analysis from linear regression.

		Freundlich Linear Fitting						
		K_F [(mg/g)/(mg/L)^n]	n	SSE	R^2	Adj. R^2	Red. χ^2	RMSE
$HF\text{-}Ti_3C_2$	SMX	0.030	0.606	0.669	0.768	0.710	0.167	0.409
	TMP	0.072	0.803	0.319	0.866	0.843	0.053	0.230
	NOR	0.153	1.203	0.299	0.748	0.712	0.043	0.207
	LEV	0.082	0.897	0.860	0.646	0.595	0.123	0.350
$HCl/HF\text{-}Ti_3C_2$	SMX	0.058	0.742	0.134	0.920	0.900	0.034	0.183
	TMP	0.119	1.004	0.183	0.883	0.860	0.037	0.191
	NOR	0.315	1.906	0.107	0.786	0.756	0.015	0.124
	LEV	0.163	1.148	0.016	0.976	0.971	0.003	0.056
$LiF/HCl\text{-}Ti_3C_2$	SMX	0.045	0.748	0.056	0.961	0.947	0.019	0.137
	TMP	0.222	1.380	0.129	0.855	0.826	0.026	0.161
	NOR	0.494	2.365	0.196	0.585	0.526	0.028	0.167
	LEV	0.364	2.009	0.123	0.754	0.714	0.021	0.143
PVDF	SMX	0.000	0.149	0.016	0.999	0.997	0.016	0.126
	TMP	0.015	0.411	0.833	0.799	0.699	0.416	0.645
	NOR	0.000	0.220	1.602	0.886	0.829	0.801	0.895
	LEV	0.002	0.279	0.906	0.889	0.833	0.453	0.673

Table S12. Summary of the Langmuir adsorption isotherm fitting parameters and statistical error analysis from nonlinear regression. Blank spaces indicate adsorption isotherms that could not be derived by nonlinear curve fitting.

		Langmuir Nonlinear fitting							
		Q_m	K_L	R_L	SSE	R^2	Adj. R^2	Red. χ^2	RMSE
HF-Ti_3C_2	SMX								
	TMP								
	NOR								
	LEV								
HCl/HF-Ti_3C_2	SMX								
	TMP	3.94337	0.0416	0.706–0.980	0.05327	0.96605	0.96039	0.00888	0.09422
	NOR	1.68447	0.19865	0.335–0.910	0.08617	0.92066	0.90743	0.01436	0.11984
	LEV	24.47871	0.00552	0.948–0.997	0.03665	0.97067	0.96578	0.00611	0.07815
LiF/HCl-Ti_3C_2	SMX	2.78432	0.03764	0.727–0.982	0.00893	0.98147	0.97684	0.00223	0.04726
	TMP	4.93051	0.03617	0.734–0.982	0.05144	0.97046	0.96554	0.00857	0.09259
	NOR	3.43076	0.12571	0.443–0.941	0.43201	0.84078	0.80894	0.0864	0.29394
	LEV								
PVDF	SMX								
	TMP								
	NOR								
	LEV								

Table S13. Summary of the Langmuir adsorption isotherm fitting parameters and statistical error analysis from linear regression.

		Langmuir Linear Fitting							
		**Q_m**	**K_L**	**R_L**	**SSE**	**R^2**	**Adj. R^2**	**Red. χ^2**	**RMSE**
**HF-Ti_3C_2**	**SMX**	0.953	0.030	0.769–0.985	2.24E+03	0.299	0.124	561.166	23.689
	**TMP**	0.833	0.100	0.501–0.953	282.939	0.542	0.466	47.157	6.867
	**NOR**	2.675	0.054	0.648–0.974	17.393	0.826	0.801	2.485	1.576
	**LEV**	0.790	0.105	0.488–0.950	265.628	0.516	0.447	37.947	6.160
**HCl/HF-Ti_3C_2**	**SMX**	−0.162	−0.192	−1.08–25.7	158.703	0.847	0.809	39.676	6.299
	**TMP**	0.612	0.266	0.273–0.882	84.980	0.536	0.443	16.996	4.123
	**NOR**	0.748	0.891	0.101–0.692	7.522	0.460	0.382	1.075	1.037
	**LEV**	2.734	0.063	0.613–0.969	0.621	0.975	0.970	0.124	0.352
**LiF/HCl-Ti_3C_2**	**SMX**	−0.223	−0.138	−2.65–3.21	55.679	0.939	0.919	18.560	4.308
	**TMP**	0.646	0.653	0.133–0.754	19.055	0.490	0.388	3.811	1.952
	**NOR**	0.980	1.197	0.077–0.626	2.114	0.544	0.478	0.302	0.550
	**LEV**	0.753	1.258	0.074–0.613	3.647	0.541	0.464	0.608	0.780
**PVDF**	**SMX**	0.000	−0.332	−1.51–1.20	4.21E+10	0.891	0.781	4.21E+10	2.05E+05
	**TMP**	−0.022	−0.207	−28.9–1.12	4.68E+03	0.794	0.691	2.34E+03	48.376
	**NOR**	0.000	−0.233	−6.10–1.13	9.94E+07	0.789	0.684	4.97E+07	7.05E+03
	**LEV**	−0.001	−0.250	−3.97–1.14	9.02E+05	0.818	0.728	4.51E+05	671.680

Table S14. Summary of the Dubinin–Radushkevich adsorption isotherm fitting parameters and statistical error analysis from nonlinear regression.

		Dubinin-Radushkevich Nonlinear fitting							
		**Q_m [mg/g]**	**β**	**E [kJ/mol]**	**SSE**	**R^2**	**Adj. R^2**	**Red. χ^2**	**RMSE**
**HF-Ti_3C_2**	**SMX**	1.125	2.578	0.440	0.047	0.956	0.947	0.010	0.097
	**TMP**	1.510	3.311	0.389	0.118	0.943	0.935	0.027	0.130
	**NOR**	1.365	3.918	0.357	0.093	0.930	0.920	0.013	0.115
	**LEV**	1.121	1.769	0.532	0.265	0.824	0.798	0.038	0.195
**HCl/HF-Ti_3C_2**	**SMX**	1.406	5.179	0.311	0.078	0.919	0.903	0.016	0.125
	**TMP**	1.217	1.747	0.535	0.030	0.981	0.977	0.005	0.071
	**NOR**	1.058	0.776	0.802	0.225	0.793	0.758	0.038	0.194
	**LEV**	1.131	1.716	0.540	0.200	0.840	0.813	0.033	0.183
**LiF/HCl-Ti_3C_2**	**SMX**	0.760	1.869	0.517	0.007	0.985	0.981	0.002	0.043
	**TMP**	1.281	1.454	0.586	0.205	0.882	0.863	0.034	0.185
	**NOR**	1.412	0.278	1.341	0.866	0.681	0.617	0.173	0.416
	**LEV**	1.199	0.924	0.736	0.897	0.570	0.509	0.128	0.358
**PVDF**	**SMX**	5.503	17.429	0.169	0.000	1.000	1.000	0.000	0.000
	**TMP**	3.894	7.776	0.254	0.186	0.961	0.949	0.062	0.249
	**NOR**	0.877	2.883	0.416	0.000	1.000	1.000	0.000	0.000
	**LEV**	3.951	8.063	0.249	0.006	0.999	0.998	0.002	0.043

Table S15. Summary of the Dubinin–Radushkevich isotherm adsorption fitting parameters and statistical analysis from linear regression.

		Dubinin-Radushkevich Linear fitting							
		Q_m [mg/g]	β	E [kJ/mol]	SSE	R^2	Adj. R^2	Red. χ^2	RMSE
$HF-Ti_3C_2$	SMX	0.545	0.993	0.710	6.653	0.565	0.456	1.663	1.290
	TMP	0.487	0.441	1.064	6.139	0.512	0.431	1.023	1.012
	NOR	0.614	0.415	1.098	2.355	0.625	0.571	0.336	0.580
	LEV	0.477	0.483	1.017	6.726	0.477	0.403	0.961	0.980
$HCl/HF-Ti_3C_2$	SMX	0.723	0.929	0.734	0.460	0.948	0.935	0.115	0.339
	TMP	0.561	0.334	1.224	4.011	0.516	0.419	0.802	0.896
	NOR	0.677	0.162	1.757	1.563	0.414	0.330	0.223	0.473
	LEV	0.779	0.591	0.920	0.707	0.793	0.751	0.141	0.376
$LiF/HCl-Ti_3C_2$	SMX	0.589	0.882	0.753	0.307	0.959	0.946	0.102	0.320
	TMP	0.650	0.205	1.562	2.654	0.441	0.329	0.531	0.729
	NOR	0.923	0.135	1.927	1.554	0.380	0.291	0.222	0.471
	LEV	0.734	0.132	1.946	1.621	0.390	0.288	0.270	0.520
PVDF	SMX	0.071	3.219	0.394	5.311	0.920	0.841	5.311	2.305
	TMP	2.530	2.407	0.456	0.574	0.974	0.961	0.287	0.536
	NOR	2.209	3.691	0.368	1.090	0.985	0.978	0.545	0.738
	LEV	1.650	2.587	0.440	0.513	0.988	0.982	0.256	0.506

Table S16. Summary of the pseudo-first order (PFO) kinetic parameters and statistical error analysis from nonlinear regression.

		Pseudo-First Order (PFO) Nonlinear fitting						
		k_1	$q_{e, calc.}$	SSE	R^2	Adj. R^2	Red. χ^2	RMSE
$HF-Ti_3C_2$	SMX	0.133	0.010	4.10E-05	0.679	0.599	1.02E-05	3.20E-03
	TMP	14.281	0.056	4.61E-03	−1.040	−1.550	1.15E-03	3.40E-02
	NOR	0.041	0.142	8.26E-03	0.674	0.593	2.07E-03	4.54E-02
	LEV	0.109	0.063	8.00E-04	0.797	0.747	2.00E-04	1.41E-02
$HCl/HF-Ti_3C_2$	SMX	0.042	0.024	1.04E-04	0.847	0.808	2.60E-05	5.10E-03
	TMP	0.039	0.061	6.17E-04	0.830	0.787	1.54E-04	1.24E-02
	NOR	0.006	0.125	2.10E-03	0.710	0.637	5.24E-04	2.29E-02
	LEV	0.065	0.040	3.15E-04	0.815	0.769	7.89E-05	8.88E-03
$LiF/HCl-Ti_3C_2$	SMX	0.055	0.022	6.17E-05	0.852	0.815	1.54E-05	3.93E-03
	TMP	0.033	0.156	1.36E-04	0.994	0.992	3.39E-05	5.82E-03
	NOR	0.006	0.527	1.15E-02	0.912	0.890	2.87E-03	5.36E-02
	LEV	0.007	0.339	1.95E-03	0.968	0.960	4.86E-04	2.21E-02
PVDF	SMX	0.017	0.001	4.27E-08	0.951	0.927	2.13E-08	1.46E-04
	TMP	0.013	0.076	7.31E-04	0.825	0.781	1.83E-04	1.35E-02
	NOR	0.175	0.107	3.93E-05	0.956	0.995	9.83E-06	3.14E-03
	LEV	0.068	0.027	6.82E-05	0.893	0.867	1.70E-05	4.13E-03

Table S17. Summary of the pseudo-second order (PSO) kinetic parameters and statistical error analysis from nonlinear regression.

		Pseudo-Second Order (PSO) Nonlinear fitting						
		k_2	$q_{e,calc.}$	SSE	R^2	Adj. R^2	Red. χ^2	RMSE
$HF\text{-}Ti_3C_2$	SMX	14.725	0.011	3.70E-05	0.710	0.638	9.25E-06	3.04E-03
	TMP	2.29E+22	0.045	5.49E-04	0.757	0.696	1.37E-04	1.17E-02
	NOR	0.205	0.172	6.95E-03	0.726	0.657	1.74E-03	4.17E-02
	LEV	2.392	0.068	7.08E-04	0.821	0.776	1.77E-04	1.33E-02
$HCl/HF\text{-}Ti_3C_2$	SMX	1.984	0.026	1.43E-04	0.789	0.737	3.56E-05	5.97E-03
	TMP	0.969	0.065	6.97E-04	0.807	0.760	1.74E-04	1.32E-02
	NOR	0.129	0.112	1.87E-03	0.741	0.676	4.68E-04	2.16E-02
	LEV	1.772	0.044	3.16E-04	0.815	0.769	7.90E-05	8.89E-03
$LiF/HCl\text{-}Ti_3C_2$	SMX	4.158	0.023	5.36E-05	0.872	0.840	1.34E-05	3.66E-03
	TMP	0.236	0.176	4.58E-04	0.979	0.973	1.15E-04	1.07E-02
	NOR	0.008	0.703	1.05E-02	0.920	0.899	2.63E-03	5.13E-02
	LEV	0.011	0.480	1.78E-03	0.971	0.964	4.46E-04	2.11E-02
PVDF	SMX	7.981	0.002	4.69E-08	0.947	0.920	2.35E-08	1.53E-04
	TMP	0.214	0.086	5.57E-04	0.867	0.833	1.39E-04	1.18E-02
	NOR	3.898	0.110	3.08E-05	0.997	0.996	7.69E-06	2.77E-03
	LEV	3.321	0.029	5.39E-05	0.916	0.895	1.35E-05	3.67E-03

Table S18. Comparison of the adsorption isotherm and kinetic parameters for the adsorption of antibiotics and dyes.

Adsorbent Material	Adsorbate	Adsorption Isotherm Parameters	Adsorption Kinetic Parameters	Removal Mechanisms	Reference
LiF/HCl-Ti_3C_2 (MXene-PVDF membrane composite)	Norfloxacin	Freundlich: K_F = 0.498 (mg/g)/(mg/L)^n; n = 1.707; Adj. R^2 = 0.869<br><br>Langmuir: Q_m = 3.43 mg/g; K_L = 0.126 L/mg; Adj. R^2 = 0.809	PFO: q_e = 0.527 mg/g; k_1 = 0.006 min^{-1}; Adj. R^2 = 0.890<br><br>PSO: q_e = 0.703 mg/g; k_2 = 0.008 g/mg-min; R^2 = 0.899<br><br>ID: k_i = 0.025 g/mg-$min^{0.5}$; C = 2E-4 mg/g ; R^2 = 0.951	Electrostatic interactions, hydrogen bonding, van der Waals interaction	This work
LiF/HCl-Ti_3C_2 (nanosheets)	Norfloxacin	Freundlich: K_F = 126.12 (mg/g)/(mg/L)^n; n = 3.156 ; Adj. R^2 = 0.926<br><br>Langmuir: Q_m = 269.20 mg/g; K_L = 0.892 L/mg; Adj. R^2 = 0.962	n.d.	Electrostatic interactions, hydrogen bonding, van der Waals interaction	This work
LiF/HCl-Ti_3C_2 (nanosheets)	Trimethoprim	Freundlich: K_F = 7.377 (mg/g)/(mg/L)^n; n = 1.292 ; Adj. R^2 = 0.937<br><br>Langmuir: Q_m = 130.554 mg/g; K_L = 0.050 L/mg; Adj. R^2 = 0.936	n.d.	Electrostatic interactions, hydrogen bonding, van der Waals interaction	This work
MXene	Methylene Red	n.d.	PFO: q_e = 61.5646 mg/g; k_1 = 0.1225 min^{-1}; R^2 = 0.7874<br><br>PSO: q_e = 69.2133 mg/g; k_2 = 0.0023 g/mg-h; R^2 = 0.8849<br><br>ID: k_i = 0.6.3752 g/mg-$h^{0.5}$; C = 15.293 mg/g ; R^2 = 0.9497	Electrostatic interactions	[3]
MXene	Methylene Blue	Freundlich: R^2 = 0.9969<br>Langmuir: R^2 = 0.9807<br>(adsorption constant values not reported)	PFO: q_e = 8.09 mg/g; k_1 = 0.0068 min^{-1}; R^2 = 0.5053	Electrostatic interactions	[4]

			PSO: q_e = 80.65 mg/g; k_2 = 0.0069 g/mg-min; R^2 = 0.9999<br><br>ID: k_i = 0.9926 g/mg-min$^{0.5}$; C = 67.66 mg/g ; R^2 = 0.4455		
MXene	Methylene Blue	Freundlich: K_F = 48.152 (mg/g)/(mg/L)^n; n = 19.963 ; R = 0.928<br><br>Langmuir: Q_m = 38.851 mg/g; K_L = 1.743 x 10^4 mL/mg; R = 0.811<br><br>(R = Pearson's coefficient)	n.d.	Electrostatic interactions	5
MXene-NH_3	Cr(VI)	Freundlich: K_F = 55.5 L/mg ; n = 6.37 ; R^2 = 0.929<br><br>Langmuir: q_m = 107.4 mg/g ; K_L = 1.416 L/g; R^2 = 0.963	PFO: q_e = 0.97.0 mg/g; k_1 = 0.02873 min^{-1}; R^2 = 0.935<br><br>PSO: q_e = 93.6 mg/g; k_2 = 0.00154 g/mg-min; R^2 = 0.838	Electrostatic interactions	6
Graphene oxide (GO)	Norfloxacin	Freundlich: K_F = 89.007 (mg/g)/(mg/L)^n; n = 2.703 ; R^2 = 0.962<br><br>Langmuir: Q_m = 334.610 mg/g; K_L = 0.238 L/mg; R^2 = 0.993	PFO: q_e = 109.917 mg/g; k_1 = 6.096 min^{-1}; R^2 = 0.988<br><br>PSO: q_e = 120.589 mg/g; k_2 = 0.299 g/mg-min; R^2 = 0.999<br><br>ID: k_i = 0.9926 g/mg-min$^{0.5}$; C = 67.66 mg/g ; R^2 = 0.4455	Van der Waals forces and π–π interaction, hydrogen bond donor and acceptor interactions	7
Multi-walled carbon nanotubes (MWCNT)	Norfloxacin	Langmuir: Q_m = 87.0 mg/g; K_L = 0.276 L/mg; R = 0.999	PSO: k_2 = 2.55 g/mg-min; R^2 > 0.99	Hydrogen bonding, electrostatic interactions, hydrophobic effect, π–π interaction	8
Polyvinyl chloride (microplastic)	Sulfamethazine	Freundlich: K_F = 46.6 L/kg ; n = 0.533 ; R^2 = 0.920	PFO: q_e = 0.0657 mg/g; k_1 = 0.032 min^{-1}; R^2 = 0.981	Electrostatic interactions, van der Waals interaction	9

		Langmuir: q_m = 107.4 mg/g ; K_L = 1.416 L/g; R^2 = 0.963	PSO: q_e = 0.0689 mg/g; k_2 = 0.833 g/mg-min; R^2 = 0.963<br><br>ID: k_i = 0.9926 g/mg-min$^{0.5}$; C = 67.66 mg/g ; R^2 = 0.4455		
Polyamide thin film composite nanofiltration membrane	Trimethoprim	Freundlich: K_F = 1.350 (ng/g)/(ng/L)^n; n = 0.668 ; R^2 = 0.999<br><br>Langmuir: Q_m = 6.313 ng/cm^2 ; K_L = 0.319 L/ng; R^2 = 0.955<br><br>Area = 80 cm^2	n.d.	Electrostatic interactions, hydrophobic	10
Polyamide thin film composite nanofiltration Membrane	Sulfamethoxazole	Freundlich: K_F = 0.114 (ng/g)/(mg/L)^n; n = 0.224 ; R^2 = 0.562<br><br>Area = 80 cm^2	n.d.	Electrostatic interaction, hydrophobic	10
Zeolite	Levofloxacin	Freundlich: K_F = 2.22-23. (mg/g)/(L/g)^n; n = 1.7-3.3 ; R^2 > 0.9041<br><br>Langmuir: Q_m = 16-45 mg/g; K_L = 0.10-1.3 L/mg; R^2 > 0.9826	n.d.		11
Attapulgite-biochar	Norfloxacin	Freundlich: K_F = 1.58 (mg/g)/(L/g)^n; n = 0.48 ; R^2 = 0.86<br><br>Langmuir: Q_m = 5.02 mg/g; K_L = 0.45 L/mg; R^2 = 0.93	PFO: q_e = 0.81 mg/g; k_1 = 2.65 min^{-1}; R^2 = 0.74<br><br>PSO: q_e = 1.98 mg/g; k_2 = 0.04 g/mg-min; R^2 = 0.99<br><br>ID: k_i = 0.009 g/mg-min$^{0.5}$; C = 1.61 mg/g ; R^2 = 0.89	Hydrophobic interactions, π–π electron–donor–acceptor (EDA) interaction, hydrogen bond formation	12

Density Functional Theory Calculations

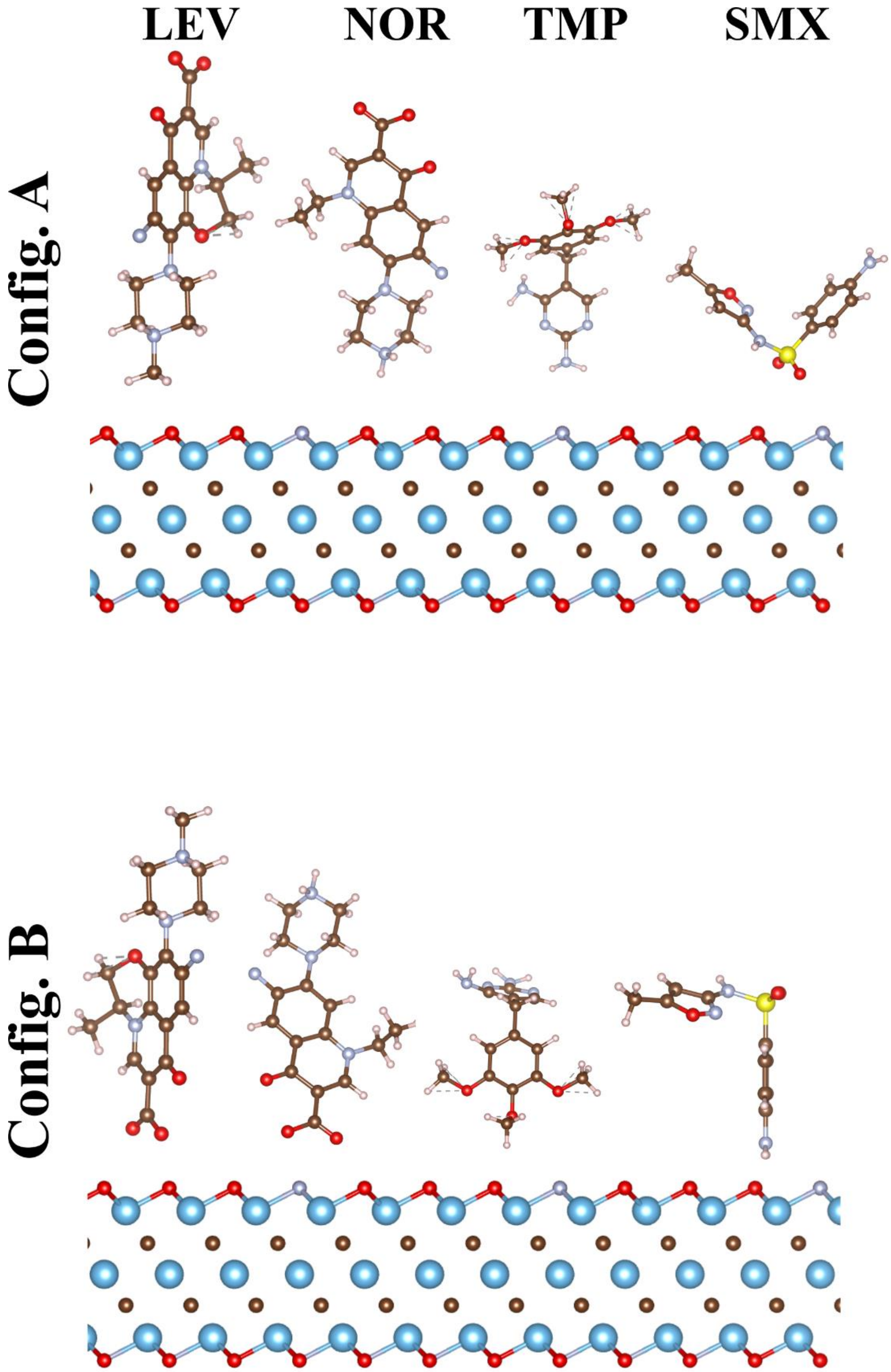

Figure S6. Schematics of antibiotics approaching the MXene surface (positioned 2Å above the surface terminations). Antibiotics are in speciation form present at neutral pH.

References

(1) *Geometrical Descriptors Plugin Chemaxon Docs*. https://docs.chemaxon.com/display/docs/geometrical-descriptors-plugin.md (accessed 2023-09-28).

(2) *Help Online - Origin Help - Theory of Nonlinear Curve Fitting*. https://www.originlab.com/doc/en/Origin-help/NLFit-theory (accessed 2023-11-26).

(3) Lim, S.; Kim, J. H.; Park, H.; Kwak, C.; Yang, J.; Kim, J.; Ryu, S. Y.; Lee, J. Role of Electrostatic Interactions in the Adsorption of Dye Molecules by Ti3C2-MXenes. *RSC Adv.* **2021**, *11* (11), 6201–6211. https://doi.org/10.1039/D0RA10876F.

(4) Jun, B.-M.; Heo, J.; Taheri-Qazvini, N.; Park, C. M.; Yoon, Y. Adsorption of Selected Dyes on Ti3C2Tx MXene and Al-Based Metal-Organic Framework. *Ceramics International* **2020**, *46* (3), 2960–2968. https://doi.org/10.1016/j.ceramint.2019.09.293.

(5) Mashtalir, O.; Cook, K. M.; Mochalin, V. N.; Crowe, M.; Barsoum, M. W.; Gogotsi, Y. Dye Adsorption and Decomposition on Two-Dimensional Titanium Carbide in Aqueous Media. *J. Mater. Chem. A* **2014**, *2* (35), 14334–14338. https://doi.org/10.1039/C4TA02638A.

(6) Kong, A.; Sun, Y.; Peng, M.; Gu, H.; Fu, Y.; Zhang, J.; Li, W. Amino-Functionalized MXenes for Efficient Removal of Cr(VI). *Colloids and Surfaces A: Physicochemical and Engineering Aspects* **2021**, *617*, 126388. https://doi.org/10.1016/j.colsurfa.2021.126388.

(7) Moreira, V. R.; Lebron, Y. A. R.; da Silva, M. M.; de Souza Santos, L. V.; Jacob, R. S.; de Vasconcelos, C. K. B.; Viana, M. M. Graphene Oxide in the Remediation of Norfloxacin from Aqueous Matrix: Simultaneous Adsorption and Degradation Process. *Environ Sci Pollut Res* **2020**, *27* (27), 34513–34528. https://doi.org/10.1007/s11356-020-09656-6.

(8) Yang, W.; Lu, Y.; Zheng, F.; Xue, X.; Li, N.; Liu, D. Adsorption Behavior and Mechanisms of Norfloxacin onto Porous Resins and Carbon Nanotube. *Chemical Engineering Journal* **2012**, *179*, 112–118. https://doi.org/10.1016/j.cej.2011.10.068.

(9) Guo, X.; Liu, Y.; Wang, J. Sorption of Sulfamethazine onto Different Types of Microplastics: A Combined Experimental and Molecular Dynamics Simulation Study. *Marine Pollution Bulletin* **2019**, *145*, 547–554. https://doi.org/10.1016/j.marpolbul.2019.06.063.

(10) Liu, Y.; Wang, X.; Yang, H.; Xie, Y. F. Adsorption of Pharmaceuticals onto Isolated Polyamide Active Layer of NF/RO Membranes. *Chemosphere* **2018**, *200*, 36–47. https://doi.org/10.1016/j.chemosphere.2018.02.088.

(11) Martucci, A.; Pasti, L.; Marchetti, N.; Cavazzini, A.; Dondi, F.; Alberti, A. Adsorption of Pharmaceuticals from Aqueous Solutions on Synthetic Zeolites. *Microporous and Mesoporous Materials* **2012**, *148* (1), 174–183. https://doi.org/10.1016/j.micromeso.2011.07.009.

(12) Li, Y.; Wang, Z.; Xie, X.; Zhu, J.; Li, R.; Qin, T. Removal of Norfloxacin from Aqueous Solution by Clay-Biochar Composite Prepared from Potato Stem and Natural Attapulgite. *Colloids and Surfaces A: Physicochemical and Engineering Aspects* **2017**, *514*, 126–136. https://doi.org/10.1016/j.colsurfa.2016.11.064.
